# The C-terminal region of Net1 is an activator of RNA polymerase I transcription with conserved features from yeast to human

**DOI:** 10.1371/journal.pgen.1008006

**Published:** 2019-02-25

**Authors:** Katharina Hannig, Virginia Babl, Kristin Hergert, Andreas Maier, Michael Pilsl, Christopher Schächner, Ulrike Stöckl, Philipp Milkereit, Herbert Tschochner, Wolfgang Seufert, Joachim Griesenbeck

**Affiliations:** Institut für Biochemie, Genetik und Mikrobiologie, Universität Regensburg, Regensburg, Germany; The University of North Carolina at Chapel Hill, UNITED STATES

## Abstract

RNA polymerase I (Pol I) synthesizes ribosomal RNA (rRNA) in all eukaryotes, accounting for the major part of transcriptional activity in proliferating cells. Although basal Pol I transcription factors have been characterized in diverse organisms, the molecular basis of the robust rRNA production *in vivo* remains largely unknown. In *S*. *cerevisiae*, the multifunctional Net1 protein was reported to stimulate Pol I transcription. We found that the Pol I-stimulating function can be attributed to the very C-terminal region (CTR) of Net1. The CTR was required for normal cell growth and Pol I recruitment to rRNA genes *in vivo* and sufficient to promote Pol I transcription *in vitro*. Similarity with the acidic tail region of mammalian Pol I transcription factor UBF, which could partly functionally substitute for the CTR, suggests conserved roles for CTR-like domains in Pol I transcription from yeast to human.

## Author summary

The production of ribosomes, cellular factories of protein synthesis, is an essential process driving proliferation and cell growth. Ribosome biogenesis is controlled at the level of synthesis of its components, ribosomal proteins and ribosomal RNA. In eukaryotes, RNA polymerase I is dedicated to transcribe the ribosomal RNA. RNA polymerase I has been identified as a potential target for cell proliferation inhibition. Here we describe the C-terminal region of Net1 as an activator of RNA polymerase I transcription in baker’s yeast. In the absence of this activator RNA polymerase I transcription is downregulated and cell proliferation is strongly impaired. Strikingly, this activator might be conserved in human cells, which points to a general mechanism. Our discovery will help to gain a better understanding of the molecular basis of ribosomal RNA synthesis and may have implications in developing strategies to control cellular growth.

## Introduction

**Fig 1:**
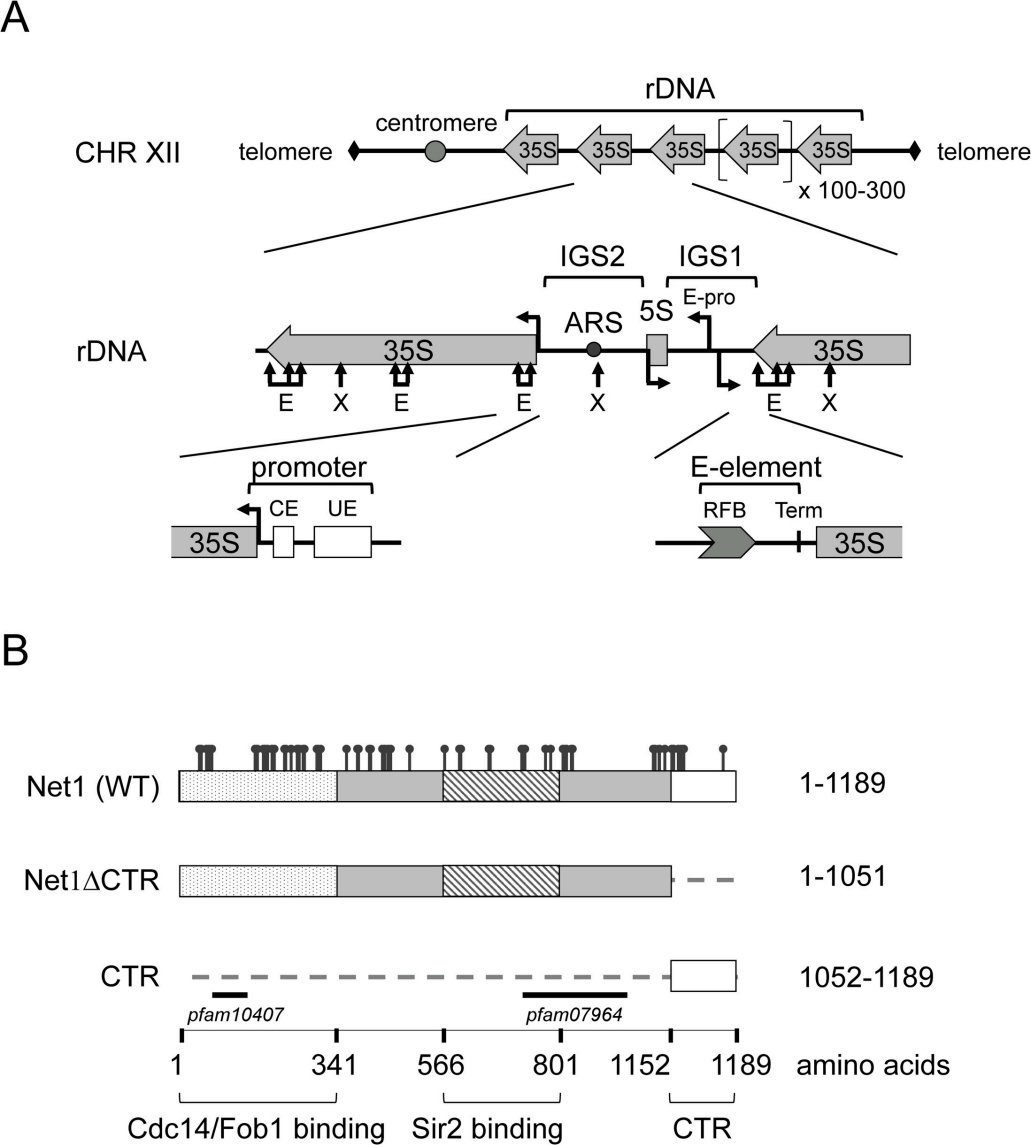
Schematic representation of the ribosomal DNA (rDNA) locus and the Net1 protein of *S*. *cerevisiae*. (A) On top: schematic representation of the yeast rDNA locus on the right arm of chromosome XII (CHR XII), consisting of 100–300 repeats (cartoon on the top). In the middle: enlargement of one rDNA repeat depicting 35S rDNA transcription units, intergenic sequences 1 and 2 (IGS1,2), the 5S rDNA, an autonomous replication sequence (ARS), and a bi-directional Pol II-dependent promoter (E-pro). Positions of restriction sites (E, EcoRI; X, XcmI) used in ChEC and psoralen crosslinking experiments are depicted. At the bottom: *cis* elements at the 5’- (promoter) and 3’- (E-element) region of the 35S rDNA, including the core element (CE), the upstream element (UE), replication fork barrier element (RFB), and the Pol I transcription termination site (Term). Arrows at promoter regions point in the direction of transcription. B) Schematic representation of the Net1 protein, and the C-terminal and N-terminal truncated version of the protein analyzed in this study (Net1ΔCTR and CTR, respectively). Amino acids included in the different proteins are depicted on the right. Different domains within the Net1 sequence interacting with Cdc14, Fob1 and Sir2 are depicted at the bottom, and represented in the cartoon as dotted, and striped rectangles, respectively. Black bars denote the positions of two *pfam* motifs found in Net1. The positions of acknowledged phosphorylation sites are shown on top of the cartoon representing the full-length Net1 protein.

RNA polymerase I (Pol I) transcribes the precursor for three out of the four ribosomal RNAs (rRNAs), which are essential components of ribosomes, required for cell growth and proliferation. In proliferating cells of *S*. *cerevisiae* (hereafter called yeast), Pol I activity accounts for over 50% of the cellular transcriptional activity [1]. Robust rRNA production is supported by repetitive gene arrays at one or multiple chromosomal locations, collectively called the ribosomal DNA (rDNA) loci (see Fig 1 upper panel, yeast rDNA). Furthermore, the specialized Pol I transcription machinery, including dedicated transcription factors, promotes efficient rRNA synthesis (reviewed in [2,3]). In the past, many factors supporting Pol I transcription *in vivo* and *in vitro* have been identified in various organisms. However, it is still an open question how the observed high transcriptional output is mechanistically achieved.

Basal mechanisms of Pol I transcription might be conserved since there appears to be a significant degree of similarity even between distantly related species. Both, mammalian and yeast promoters are arranged in two main domains including a core element (CE, Fig 1A), required for transcription initiation *in vitro*, and an upstream (control) element (UE in yeast (Fig 1A), UCE in mammals), supporting activated transcription under certain conditions (reviewed in [3]). Whereas promoter DNA sequences are unrelated, components of CE-binding complexes, the yeast core factor (CF) and the human Selectivity Factor 1 (SL1) share functional and structural homology [4–7]. CF and SL1 are required to recruit the conserved initiation competent Rrn3-Pol I complex to the rDNA promoter [8,9]. Stable recruitment of CF to the rDNA promoter in yeast depends on the 6-subunit upstream activation factor (UAF) binding to the UE [10]. In analogy, the high mobility group (HMG)-box protein upstream binding factor (UBF) interacts with the UCE and stabilizes the SL1 complex at mammalian rDNA promoters [11,12]. UBF has, however, no reported homology with UAF components. Apart its function in Pol I pre-initiation complex (PIC) formation, UBF can bind the entire rDNA region transcribed by Pol I presumably to maintain an open chromatin structure [13,14]. In yeast, the HMG-box protein Hmo1 is a component of open rDNA chromatin [15,16], and genetic data suggests functional conservation between distinct HMG-boxes in human UBF and Hmo1 [17].

Regulation of Pol I transcription by post-translational covalent modifications of proteins has been extensively described and characterized in mammals (reviewed in [18,19]). Thus, the polymerase, Rrn3, SL1 and UBF are targets of phosphorylation or acetylation, and many factors either attaching or removing specific modifications marks have been reported. Post-translational modifications occur in response to intra- or extracellular signals mainly implicated in the regulation of cell growth and proliferation. Like in higher eukaryotes, yeast ribosome biogenesis responds to different growth conditions (reviewed in [20]). However, for this organism detailed knowledge about post-translational modifications regulating the activity of the basal Pol I transcription machinery is rather limited. Recently, yeast phosphatase Cdc14, the orthologue of mammalian Cdc14B, has been proposed to downregulate Pol I transcription by de-phosphorylation of a polymerase subunit in late anaphase of the cell cycle [21]. This differs from observations in higher eukaryotes, where Cdc14B phosphatase activity is required for re-activation of Pol I transcription at the end of mitosis [22]. Likewise, both, the yeast NAD-dependent lysine deacetylase Sir2, and its mammalian homologue SIRT1 have been differentially implicated in the regulation of rDNA transcription. While the mammalian SIRT1 protein deacetylates components of the Pol I transcription machinery [23], yeast Sir2 rather targets RNA polymerase II (Pol II) transcription in rDNA repeats likely by acting on acetylated histones [24,25]. Therefore, although post-translational covalent modifications by homologous factors might influence rDNA transcription in yeast and mammals, it is unclear if the regulation by the respective modification marks is conserved.

In yeast, Cdc14 and Sir2 associate together with the nucleolar protein Net1, forming the “REgulator of Nucleolar silencing and Telophase” (RENT) complex [26–28]. Cdc14 and Sir2 interaction interfaces have been mapped to the N-terminal and central parts of full-length Net1, respectively (Fig 1B) [29–31]. By interacting with Cdc14, Net1 inhibits its phosphatase activity and sequesters the protein in the nucleolus. Presumably triggered by specific phosphorylation events Net1 releases Cdc14 in late anaphase. Cdc14 release in turn, leads to de-phosphorylation of nuclear and cytoplasmic substrates, which facilitate completion of anaphase, cytokinesis, and progression into G1 [32–36]. Net1 acts likely as a recruiting factor for Sir2, to establish rDNA silencing of Pol II transcription within the intergenic sequence 1 and 2 (IGS1+2, Fig 1A, middle panel, also named NTS1+2, for non-transcribed spacers 1 and 2, in the literature) [27,37–39]. Accordingly, main association sites for the RENT complex have been mapped to the 35S rDNA promoter region in IGS2, and to the replication fork barrier sequence (RFB) in IGS1 at the 3’ end of the 35S rRNA gene (Fig 1A) [37]. RENT association with the RFB depends on the fork blocking protein Fob1, presumably through interaction with the Net1 N-terminus [37,40]. Efficient association with the 35S rDNA promoter, instead, requires UAF [41].

A presumably RENT complex-independent function of Net1 in activating Pol I transcription was reported more than 15 years ago [42]. Thus, temperature sensitivity of *net1* mutants, in which rRNA production was impaired, could be suppressed by overexpression of the Pol I initiation factor Rrn3. Additionally, recombinant purified Net1 stimulated Pol I transcription *in vitro*. The molecular basis for these observations, however, remains far from being understood. In this study, we aimed at investigating how Net1 stimulates Pol I transcription. Strikingly, we discovered that the C-terminal 138 amino acids of Net1 harbored the Pol I transcription stimulating function. The C-terminal region (CTR) activated Pol I transcription outside the context of the full-length Net1 protein *in vivo* and was sufficient to stimulate Pol I transcription *in vitro*. The identification of the CTR of yeast Net1 as a Pol I transcription activator made it possible to discover similarities of this protein domain with the acidic tail region of the human Pol I transcription factor UBF. Our results are discussed in the light of apparent conservation of the Pol I transcription machineries in distantly related organisms.

## Results

### Deletion of the C-terminal 138 amino acids of Net1 significantly impairs growth

**Fig 2:**
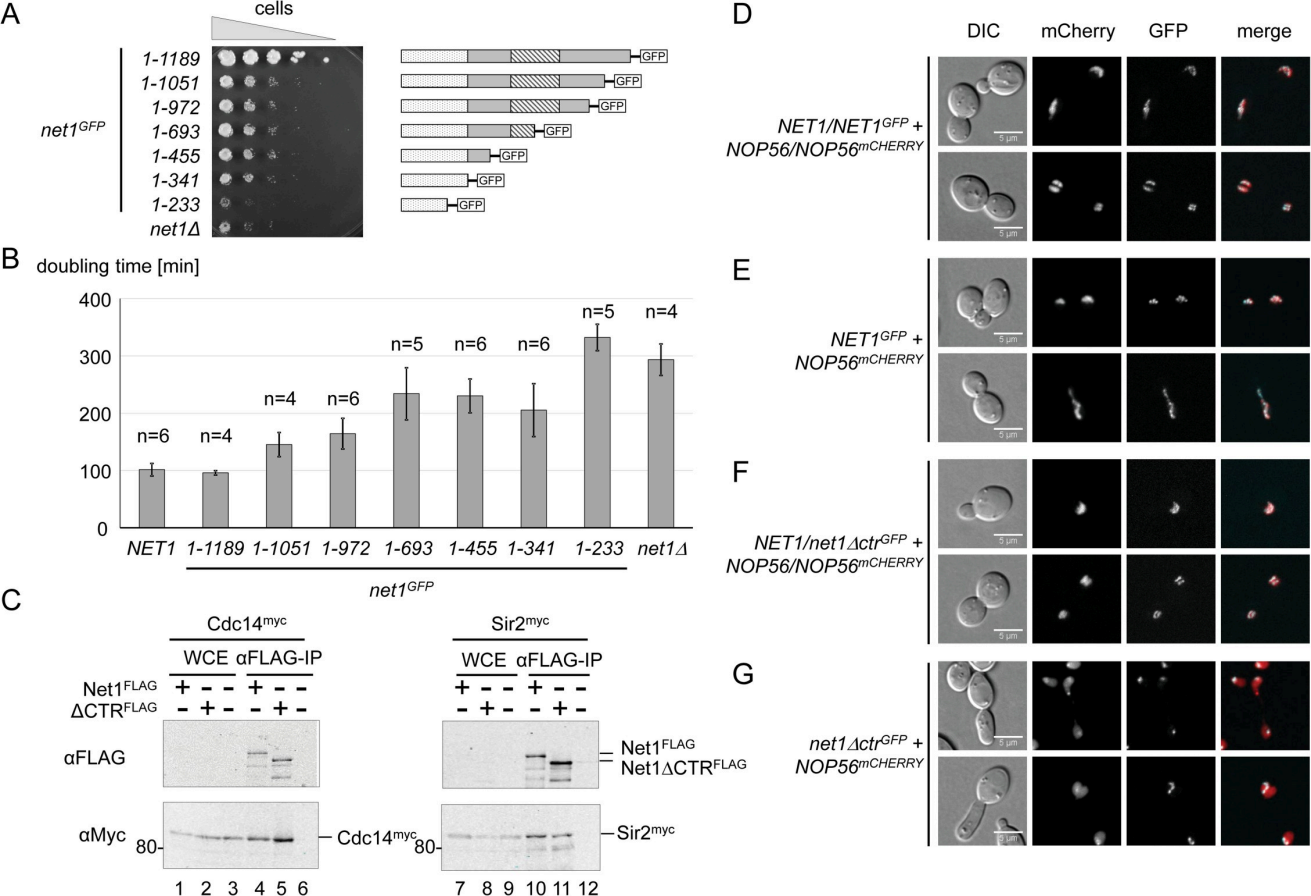
Yeast strains expressing Net1 with a C-terminal deletion are affected in growth but produce a nucleolar protein which still interacts with Cdc14 and Sir2. (A, B) Diploid yeast strains (W13533, W13534, W13535, W13536, W13537, W13538, W13539, W11979) were sporulated yielding haploid progenies carrying a deletion of *NET1* (*net1Δ*), or alleles for expression of (truncated) Net1^GFP^ fusion proteins encompassing the amino acids of the full-length Net1 as indicated on the left. All progenies expressed Nop56^mCherry^ fusion protein as nucleolar marker. A) Serial dilutions of cell suspensions of the haploid progenies of the diploid strains were spotted on XYD plates and incubated at 25°C for 2d before a photograph was taken. Cartoons of the respective Net1^GFP^ proteins are depicted on the right according to Fig 1B. B) Growth of individual haploid progenies of the diploid strains in YPD media at 30°C was determined using a TECAN plate reader system. Haploid wildtype strains y3290 and y3298 were cultured in parallel (*NET1*). The mean doubling time and standard deviation error was calculated from two to three independent biological and two independent technical replicates. The number (n) of independent measurements for the respective genotype is indicated on top of the bars. See S1 Dataset for raw data. C) Diploid yeast strains (W13776, W13777, W13778, W13779) were sporulated yielding haploid progenies expressing either Cdc14^myc^ or Sir2^myc^ and the indicated Net1^FLAG^, Net1ΔCTR^FLAG^, or the untagged wild-type Net1 protein. Whole-cell extracts (WCE) of the haploid strains were subjected to immuno-precipitation (IP) with anti-FLAG M2 antibody agarose (αFLAG-IP). Proteins were analyzed in a western blot with anti-Myc and anti-FLAG antibodies (αMyc,αFLAG). A fluorograph of the western blot membrane is shown. The size of molecular weight markers in kDa is indicated on the left. Positions of the tagged proteins are indicated on the right. D-E) Diploid yeast strains (W13533, W13534) were sporulated, yielding haploid progenies expressing Nop56^mCherry^ and either Net1^GFP^, or Net1Δctr^GFP^. Diploid parental strains (D, F) and haploid progenies (E, G) were subjected to live cell fluorescence microscopy. Data was collected for differential interference contrast (DIC, first lane of panels), mCherry, and GFP fluorescence (second, and third lane of panels, respectively). mCherry and GFP signals were merged in the fourth lane of panels. A scale bar (5μm) is shown in the first lane of panels.

We established haploid yeast strains, expressing a series of Net1 truncation mutants C-terminally fused to GFP from the endogenous *NET1* locus (Fig 2A, cartoon on the right, S1A Fig, western blot analysis). These yeast strains were subjected to growth analyses on solid medium and in liquid cultures (Fig 2A and 2B; S1 Dataset). A strain expressing full-length Net1 protein fused to GFP grew as well as a *NET1* wild-type strain (Fig 2B and S1B Fig, compare *NET1* with *net*^*-GFP*^*(1–1189)*), indicating that the GFP tag likely did not interfere with important functions of the protein. C-terminal truncation of the Net1 sequence by only 138 amino acids led to a significant slow-growth phenotype (Fig 2A and 2B, compare *net1*^*GFP*^(*1–1051)* with *net*^*-GFP*^*(1–1189)*). Additional truncation affecting the integrity of the Sir2-binding domain of Net1 increased the doubling time (Fig 2B, *net1*^*GFP*^*(1–693)*, (*1–455)*, and (*1–341)*). A strong growth defect, indistinguishable from that of a *NET1* deletion strain, was observed when the C-terminal truncation affected the Cdc14-binding domain (Fig 2A and 2B, compare *net1*^*GFP*^*(1–233)* with *net1Δ*). This suggested that the last 138 amino acids of Net1 (hereafter referred to as C-terminal region, CTR) harbor an important growth-supporting function.

### A CTR deletion mutant still associates with Cdc14 and Sir2, and localizes to the nucleolus

A mutant Net1 protein lacking the CTR (hereafter referred to as Net1ΔCTR) still contains the regions important for Net1 binding to Cdc14 and Sir2 in the context of the RENT complex (Fig 1B) [29–31]. In good agreement, both FLAG-tagged full-length Net1 (Net1^FLAG^) and Net1ΔCTR (Net1ΔCTR^FLAG^) fusion proteins co-precipitated Myc-epitope-tagged Cdc14 and Sir2 (Cdc14^myc^, Sir2^myc^) with similar efficiencies from whole-cell extracts (Fig 2C, compare lanes 4 with 5, and 10 with 11; S1C Fig, full membrane). As expected, FLAG antibody-mediated precipitation of Myc-tagged Cdc14, or Sir2, was not observed when whole-cell extracts were prepared from strains that did not express FLAG fusion protein (Fig 2C, lanes 6 and 12). We then examined if the CTR was required for the reported nucleolar localization of Net1 [26–28]. Live cell imaging of haploid and diploid yeast strains co-expressing Net1^GFP^ fusion proteins and the nucleolar marker protein Nop56 fused to mCherry (Nop56^mCherry^) confirmed co-localization of both proteins in the crescent shaped yeast nucleolus (Fig 2D and 2E). Net1ΔCTR^GFP^ showed clear nucleolar localization in a diploid yeast strain expressing a *NET1* wild-type allele (Fig 2F). This strain was not compromised in growth (not shown), indicating that the *net1Δctr*^*GFP*^ allele had no dominant negative effect. A haploid strain expressing Net1ΔCTR^GFP^ was impaired in growth (Fig 2A and 2B), and showed abnormal cell morphology (Fig 2G, DIC), and de-localization of Nop56^mCherry^ over the whole nucleus (Fig 2G, mCherry). However, a subpopulation of Nop56^mCherry^ preferentially co-localized with Net1ΔCTR^GFP^ within a subnuclear compartment, likely the remainder of the nucleolus (Fig 2G, GFP and merge).

### Expression of the CTR in *trans* rescues growth defects in *net1*Δ*ctr* and *net1*Δ strains

**Fig 3:**
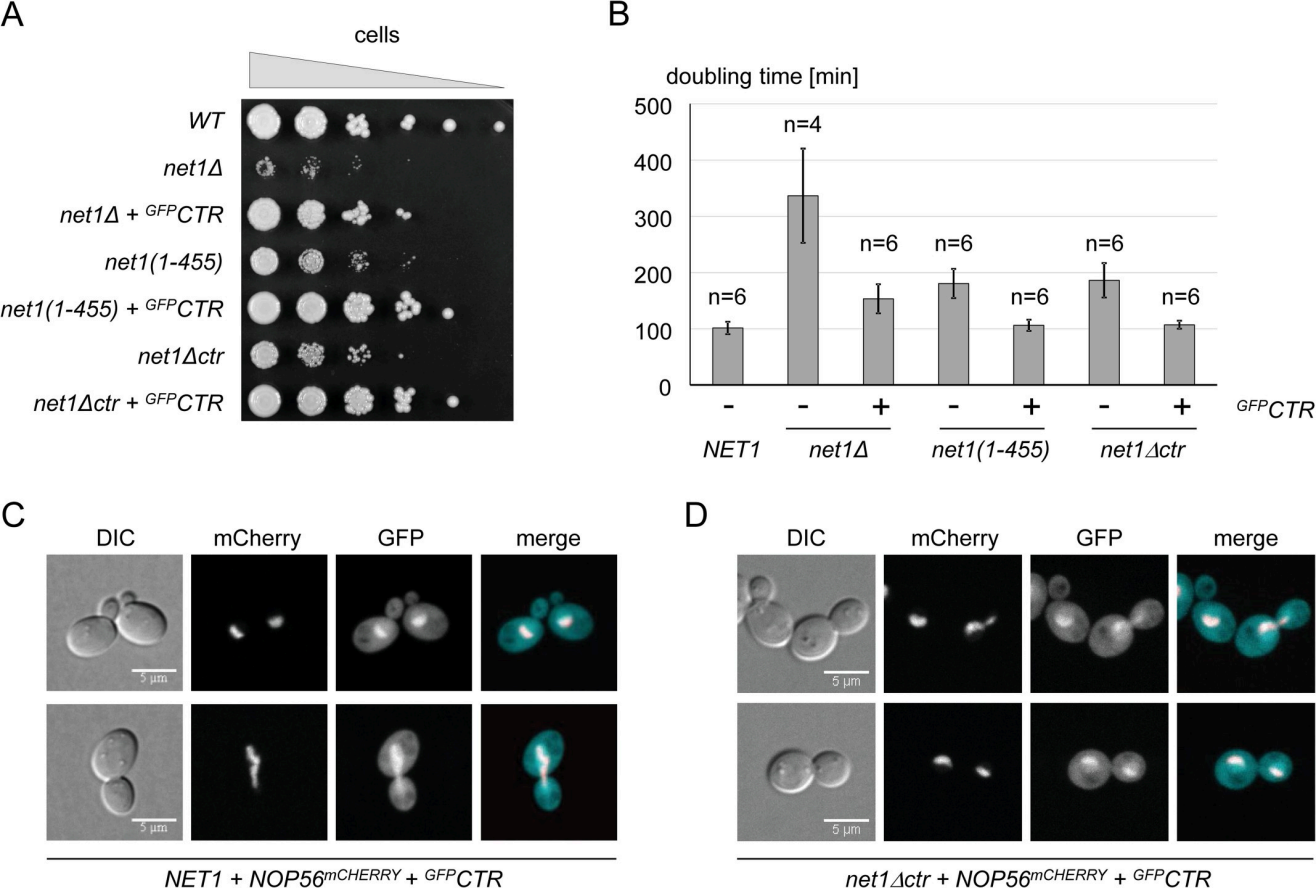
Expression of the CTR in *trans* rescues growth defects and Nop56 delocalization in *net1Δctr* strains. (A, B) Diploid yeast strains (W12509, W12533, W13762) were sporulated. Haploid progenies carried a deletion of *NET1* (*net1Δ*), or alleles for expression of the indicated truncated Net1 proteins. Where indicated (+) strains co-expressed chromosomally encoded ^GFP^CTR. Growth analyses on plates and in liquid culture were performed with individual haploid progenies of the diploid strains as described in the legend to Fig 2A and 2B. Yeast strains carrying a *NET1* wild-type allele (WT, K699 (A), and y3290 and y3298 (B)) were included as controls. C, D) The diploid yeast strain W15407 was sporulated, yielding haploid progenies carrying either a *NET1* wild-type allele (C) or a *net1Δctr* allele (D), and expressing Nop56^mCherry^ and ^GFP^CTR. These haploid strains were subjected to live cell fluorescence microscopy as described in the legend to Fig 2D.

To analyze if the CTR is an independent functional domain, we tested whether expression of the CTR in *trans* could rescue the phenotypes observed in a *net1*Δ*ctr* strain. A cassette for constitutive overexpression of the CTR N-terminally fused to GFP (^*GFP*^*CTR*) under the control of the *TEF2* promoter was stably integrated in the *LEU2* locus. Co-expression of ^*GFP*^*CTR* in *trans* fully restored wild-type growth in *net1*Δ*ctr* and *net1(1–455)* strains, and largely suppressed the growth defect of a *net1*Δ strain (Fig 3A and 3B; S1 Dataset). This indicated that the CTR and the first 455 amino acids of Net1, containing the Cdc14 binding region (but lacking the Sir2 interacting domain) are sufficient to promote normal cell growth. Live cell imaging showed a preferential nucleolar localization of ^GFP^CTR in all strains, although the overexpressed fusion protein also spread all over the cell (Fig 3C and 3D; S2B and S2C Fig, compare panels mCherry and GFP). In *net1(1–455)* and *net1*Δ*ctr* strains, overexpression of ^GFP^CTR restored normal cell morphology as well as nucleolar localization of Nop56^mCherry^ (Figs 2G and 3D; S2A and S2B Fig compare panels DIC, and mCherry, respectively). However, cellular morphology was still significantly altered in a *net1*Δ strain overexpressing ^GFP^CTR, in good correlation with the residual growth defect (S2C Fig, DIC).

As observed for expression of ^GFP^CTR, expressing the CTR in fusion with a N-terminal FLAG-tag (^FLAG^CTR) from a chromosomally integrated cassette suppressed the growth defect of *net1*Δ*ctr* strains (S2D Fig; S1 Dataset). Overexpression of the CTR did not affect the expression levels of Net1ΔCTR (S2E Fig).

### Deletion of the CTR impairs Net1 association with the 35S rDNA promoter

**Fig 4:**
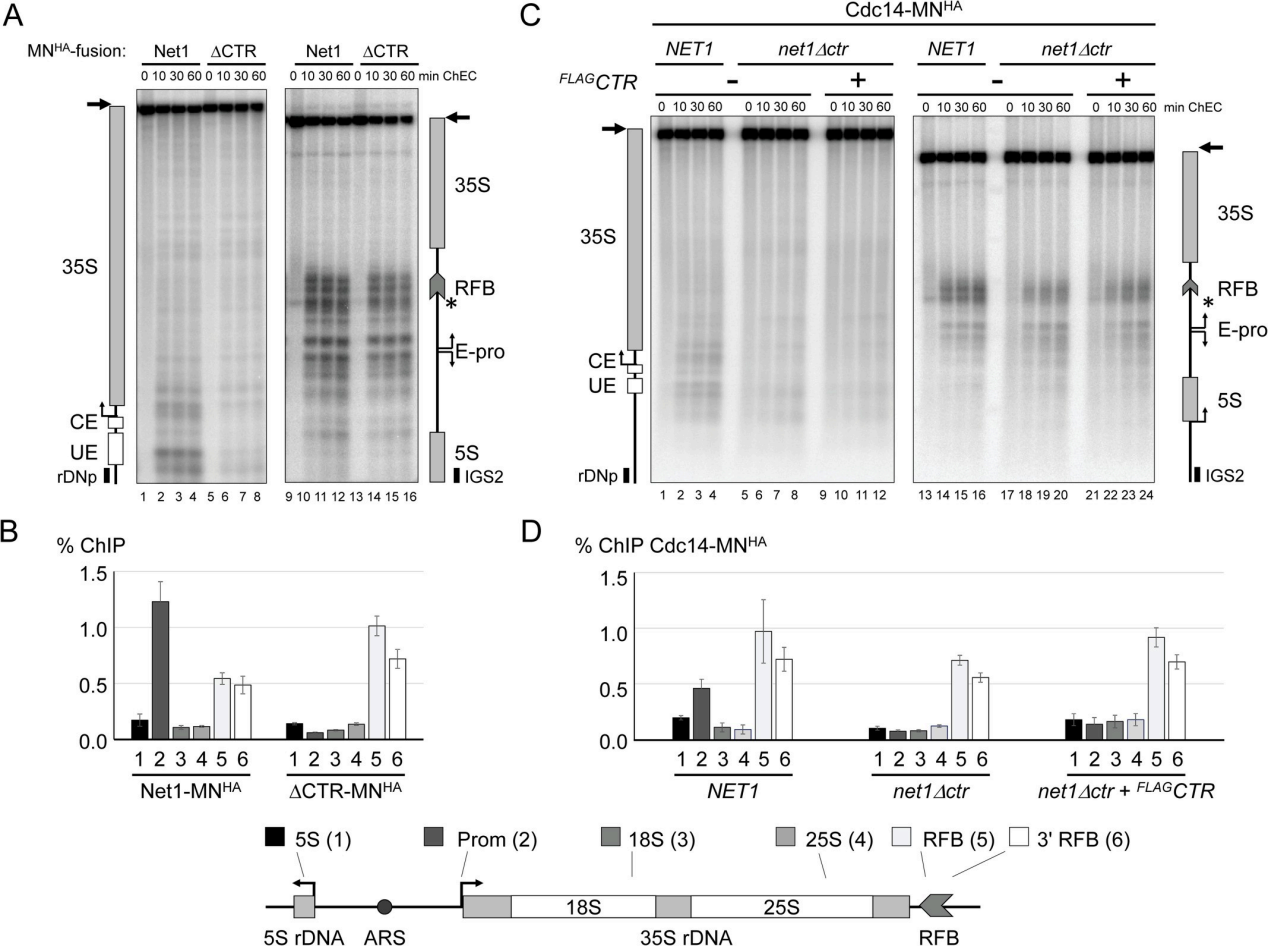
35S rDNA promoter association of RENT complex components is impaired in *net1Δctr* strains. Haploid yeast strains expressing the indicated proteins C-terminally fused to MN^HA^ were subjected to ChEC, or ChIP experiments. A, C) Crude nuclei suspensions were subjected to ChEC in the presence of calcium for the indicated times (min ChEC), DNA was isolated, cut with XcmI, separated in a 1% agarose gel and subjected to Southern blot analysis by indirect end-labeling using the radiolabeled probes rDNp, IGS2. An autoradiograph of the membrane is shown. Cartoons at the sites depict a map of the detected genomic fragments including probe hybridization sites and important *cis* elements described in the legend to Fig 1A. Arrows at the top of the cartoons point to the uncut full-length XcmI fragments. An asterisk marks the site of a DNA double-strand break occurring at the 3’ end of the RFB in the absence of ChEC. B, D) fragmented chromatin was subjected to ChIP with the indicated MN^HA^ tagged fusion proteins using anti-HA (3F10) antibody. DNA co-precipitating with the tagged proteins and from input fractions was isolated and analyzed by quantitative PCR with different primer pairs amplifying distinct regions (1–6) within the rDNA locus indicated in the cartoon at the bottom. Bar graphs depict the efficiency of co-precipitation of the fragments as percentages of the input DNA (% ChIP). Mean values and standard deviation errors were derived from three independent ChIP experiments analyzed each in triplicate qPCR reactions (n = 9). A, B) ChEC and ChIP analyses with strains (y3041, y3042, y3043, y3044) expressing either Net1-MN^HA^ or Net1ΔCTR-MN^HA^ (ΔCTR-MN^HA^). C, D) ChEC and ChIP analyses with strains (y3058, y3068, y3250), carrying a *NET1* or a *net1Δctr* allele, and expressing Cdc14-MN^HA^, in the absence or presence of a chromosomally integrated ^*FLAG*^*CTR* expression cassette.

Net1 and other RENT components associate with two rDNA regions, the 35S rDNA promoter region and the RFB at the 3’-end of the 35S rRNA gene [37]. To investigate if deletion of the CTR influenced this binding pattern, we performed Chromatin Endogenous Cleavage (ChEC) [43] and Chromatin Immuno-Precipitation (ChIP) analyses. Haploid yeast strains expressing either wild-type Net1 or Net1ΔCTR in fusion with micrococcal nuclease (MN) and a hemagglutinin (HA) epitope (Net1-MN^HA^, ΔCTR-MN^HA^) were created. For ChEC analyses, crude nuclei isolated from exponentially growing formaldehyde-crosslinked cells were treated with calcium to activate the MN. Cleavage events of the MN^HA^-fusion proteins were mapped to the indicated genomic regions by Southern blot analysis using the indirect end-labeling technique [44]. Net1-MN^HA^ fusion protein mediated cleavages were observed both at the 35S rDNA promoter and at the RFB in good correlation with earlier results (Fig 4A, lanes 1–4 and 9–12) [41]. Net1ΔCTR-MN^HA^-mediated cuts were strongly reduced at the 35S rDNA promoter region, whereas robust cleavages were still observed at the RFB (Fig 4A, lanes 5–8 and 13–16). In good agreement, Net1-MN^HA^ but not Net1ΔCTR-MN^HA^ co-precipitated 35S rDNA promoter fragments from cellular extracts in ChIP experiments (Fig 4B, compare ChIP of fragment 2, cartoon at the bottom for location of ChIP fragments in yeast rDNA). In contrast, co-precipitation of RFB fragments with Net1ΔCTR-MN^HA^ was unaltered or even slightly enhanced, when compared to ChIP with Net1-MN^HA^ (Fig 4B, compare ChIP of fragments 5, 6). As expected, no significant co-precipitation of rDNA fragments bearing the 5S rDNA, 18S rDNA, and 25S rDNA regions with the fusion proteins was observed (Fig 4B, ChIP of fragments 1, 3, 4). Thus, it can be concluded that the CTR is required for interaction of Net1 with the 35S rDNA promoter but does not affect Net1 association with the RFB, which is mediated by Fob1 [37]. In good correlation, Fob1 association with the RFB was unaltered or even slightly increased in *net1*Δ*ctr* strains when compared with *NET1* strains (S3A and S3B Fig, compare lanes 9–12 with 13–15, compare ChIP of fragments 5, 6).

### 35S rDNA promoter association of RENT complex components is impaired in the *net1*Δ*ctr* strain

Cdc14 and Sir2 have rDNA association patterns very similar to that of Net1 [37,41,45]. Accordingly, Cdc14-MN^HA^ mediated ChEC in a *NET1* wildtype strain led to cuts within the 35S rDNA promoter region and at the RFB, in good correlation with enrichment of these regions in ChIP experiments (Fig 4C, lanes 1–4, and 13–16; Fig 4D, ChIP of fragments 2, 5, 6). When expressed in a *net1*Δ*ctr* strain, Cdc14-MN^HA^-mediated cleavages at the 35S rDNA promoter after ChEC were strongly reduced and the co-precipitation of this region in ChIP experiments was abolished (Fig 4C, lanes 5–8; Fig 4D, ChIP of fragment 2). In contrast, ChEC and ChIP indicated that the interaction of Cdc14 with the RFB was very similar to that observed in the *NET1* strain (Fig 4C, lanes 17–20; Fig 4D, ChIP of fragments 5, 6). As for Cdc14-MN^HA^, Sir2-MN^HA^-mediated cleavages within the 35S rDNA promoter region in a *net1*Δ*ctr* strain were reduced when compared to cleavage events observed in a *NET1* strain (S3C Fig, upper panel, compare lanes 1–4 with 5–8). As opposed to Cdc14-MN^HA^, however, Sir2-MN^HA^ mediated cuts at the RFB in a *net1*Δ*ctr* strain were reduced, when compared with cleavages observed in a *NET1* strain (S3C Fig, lower panel, compare lanes1-4 with 5–8).

### Expression of the CTR in *trans* does not rescue 35S rDNA promoter interaction of RENT complex components in the *net1*Δ*ctr* strain

The impaired association of RENT complex components with the 35S rDNA promoter could be causative for the growth defect observed in the *net1*Δ*ctr* strain. Ectopic expression of ^FLAG^CTR in *net1*Δ*ctr* strains restored wild-type growth (Fig 3A and 3B). Thus, it was tested if the interaction of Cdc14 with the 35S rDNA promoter could be re-established in this condition. ChEC and ChIP experiments were carried out in a *net1*Δ*ctr* strain expressing Cdc14-MN^HA^ from the endogenous locus and the ^FLAG^CTR from a chromosomally integrated cassette. Expression of ^FLAG^CTR resulted neither in significant Cdc14-MN^HA^-mediated cleavage at the 35S rDNA promoter in ChEC experiments, nor did it lead to co-precipitation of 35S rDNA promoter fragments in ChIP (Fig 4C, lanes 9–12; Fig 4D, ChIP of fragment 2). Co-expression of ^FLAG^CTR did not affect Cdc14-MN^HA^ association with the RFB (Fig 4C, lanes 21–24; Fig 4D, ChIP of fragments 5, 6). In agreement with the assumption that Cdc14 is recruited to the rDNA via its interaction with the N-terminus of Net1, very similar results were obtained in ChEC experiments with a ^*FLAG*^*CTR* strain expressing Net1ΔCTR-MN^HA^ (S3D Fig, lanes 5–8).

### Pol I association with 35S rRNA genes requires the CTR

**Fig 5:**
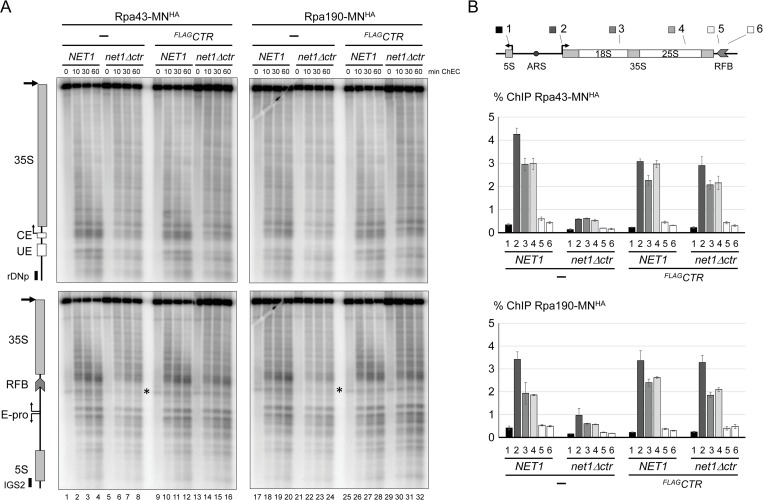
**Robust Pol I association with 35S rRNA genes requires the CTR.** (A, B) Haploid yeast strains (y3288, y3289, y3299, y3302, y3595, y3597, y3599, y3601) carrying a *NET1* or a *net1Δctr* allele, and expressing Rpa43-MN^HA^ or Rpa190-MN^HA^ in the absence or presence of the Net1 ^FLAG^CTR were subjected to ChEC (A) and ChIP (B) analyses as described in the legend to Fig 4.

The above experiments suggested, that the CTR plays a RENT complex-independent role supporting cellular growth, presumably by interacting with the 35S rDNA promoter. We therefore tested if the CTR harbors the Pol I transcription stimulating function of Net1. Haploid yeast strains carrying a *NET1* or a *net1*Δ*ctr* allele and co-expressing MN^HA^ fusion proteins of the Pol I subunits Rpa43 or Rpa190 were subjected to ChEC and ChIP experiments. ChEC analyses revealed that Rpa43-MN^HA^ and Rpa190-MN^HA^ cleavage events at the 35S rDNA promoter region and at the 35S rDNA transcription termination site upstream of the RFB were reduced in *net1*Δ*ctr* strains, when compared to cleavages in *NET1* strains (Fig 5A compare lanes 1–4 with lanes 5–8, and lanes 17–20 with lanes 21–24). This pointed to a lower occupancy of Pol I molecules within the 35S rRNA gene region. ChIP experiments strongly supported this finding, since co-precipitation of 35S rDNA fragments with the tagged Pol I subunits was substantially impaired in *net1*Δ*ctr* strains when compared with ChIP in *NET1* strains (Fig 5B, ChIP of fragments 2–4). Importantly, ChEC and ChIP experiments indicated that expression of ^FLAG^CTR in *trans* could fully restore Pol I association with the 35S rDNA in *net1*Δ*ctr* cells to wild-type levels, in good correlation with the re-establishment of normal cell growth in these strains (Fig 5A, compare lanes 9–12, and 25–28 with lanes 13–16, and 29–32, respectively; Fig 5B, ChIP of fragments 2–4; S3 Table, doubling times).

### The CTR interacts with many chromosomal loci

**Fig 6:**
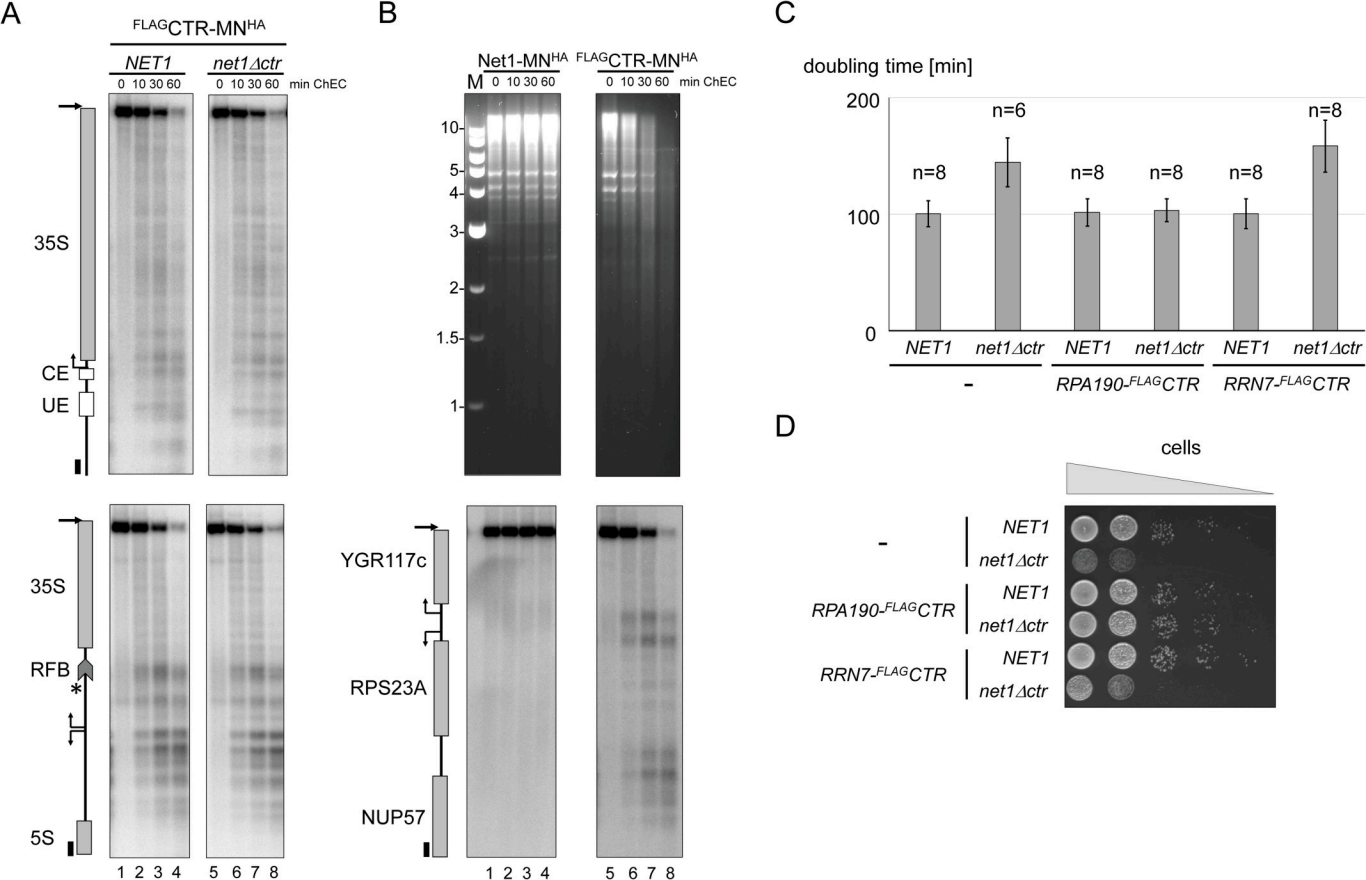
**The CTR of Net1 interacts with many chromosomal loci and rescues slow growth in *net1Δctr* strains when fused to Rpa190.** (A, B) Haploid yeast strains (y3087; y3157; y3160) carrying a *NET1* or a *net1Δctr* allele and expressing CTR-MN^HA^ or Net1-MN^HA^ were subjected to ChEC analyses, as described in the legend to Fig 4. B) Agarose gels were stained with SYBR safe before transfer to the blotting membrane (upper panels), sizes of selected DNA fragments in the DNA marker (M) are given on the left. Lower panels, the membrane was hybridized with a radioactively labeled probe hybridizing with a XcmI fragment containing the *NUP57*/*RPS23A* loci as indicated in the cartoon on the left. C, D) Haploid yeast strains (y3673; y3674; y3746; y3747; y3748; y3749; y3756; y3757; y3758; y3759, y3290, y3298) carrying a *NET1* or a *net1Δctr* allele and expressing CTR fusion proteins of Rpa190, or Rrn7 or the wild-type proteins, were subjected to growth analyses in liquid cultures (C), and on solid medium (D) as described in the legend to Fig 2A and 2B.

Since the CTR is required to recruit full-length Net1 to the 35S rDNA promoter (Fig 4A), we further investigated if the isolated CTR expressed in *trans* may interact with this chromosomal region. ChEC experiments were performed in *NET1* or *net1Δctr* strains, expressing the CTR with an N-terminal FLAG tag and C-terminally fused to MN^HA^ (^FLAG^CTR-MN^HA^), under the control of the *TEF2* promoter from a cassette integrated in the *LEU2* locus. As observed for ^GFP^CTR and ^FLAG^CTR, expression of the ^FLAG^CTR-MN^HA^ fusion protein suppressed the slow-growth phenotype of *net1Δctr* strains (S3 Table). Similar ^FLAG^CTR-MN^HA^-mediated cleavage events within the 35S rDNA promoter region were observed in both *NET1* and *net1Δctr* strains (Fig 6A, upper panels, lanes 1–4, and 5–8). ^FLAG^CTR-MN^HA^-mediated cuts were also observed at the RFB and at further sites within IGS1 (Fig 6A, lower panels, lanes 1–4, and 5–8). Additionally, ^FLAG^CTR-MN^HA^-dependent cleavages were observed at accessible sites within intergenic sequences at other genomic loci, which were not cut by Net1-MN^HA^ (Fig 6B, lower panels, lanes 1–4, and 5–12). In accordance with the observed nuclear localization of ^GFP^CTR (Fig 3C and 3D; S2B and S2C Fig), ChEC in the ^FLAG^CTR-MN^HA^-expressing strains eventually resulted in the complete degradation of genomic DNA, which did not occur in a Net1-MN^HA^-expressing strain (Fig 6B, upper panels compare lanes 1–4 with lanes 5–12). Thus, upon overexpression of the CTR, the protein is available for DNA interactions within the entire nucleus, including the 35S rDNA promoter.

### Expression of a Rpa190-CTR fusion protein suppresses the growth defect of a *net1Δctr* strain

The above data are compatible with a model in which the CTR assists Pol I recruitment at the 35S rDNA promoter. To further test this hypothesis experimentally, we fused the ^FLAG^CTR to the C-terminus of Rpa190 and the CF component Rrn7 in *net1Δctr* strains. Whereas expression of Rrn7-^FLAG^CTR could not significantly rescue the growth phenotype of *net1Δctr* strains, expression of Rpa190-^FLAG^CTR fully suppressed slow growth (Fig 6C and 6D; S1 Dataset). The expression of CTR fusion proteins had no impact on growth in *NET1* strains. This observation is in accordance with a Pol I-specific function of the CTR, but also indicates that simple tethering of the CTR to the 35S rDNA promoter may not be sufficient to obtain full stimulation of Pol I transcription.

### Pol I associates with phosphorylated CTR

**Fig 7:**
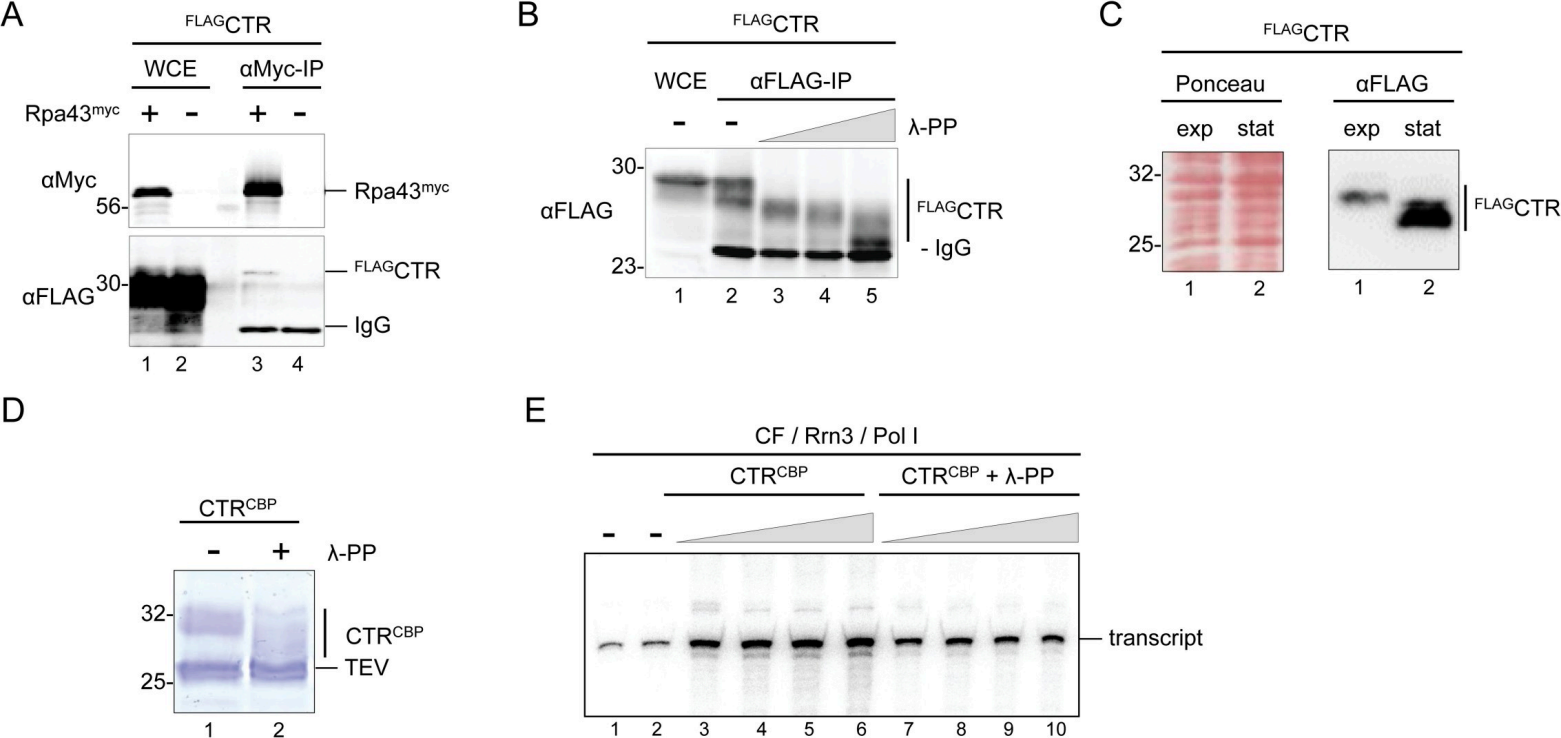
**The CTR is differentially phosphorylated in different growth states *in vivo* and CTR-dependent stimulation of Pol I transcription *in vitro* is decreased upon λ-protein phosphatase treatment.** (A) The CTR associates with Pol I. Diploid yeast strains (W12903, W13491) were sporulated yielding haploid progenies expressing ^FLAG^CTR and the wild-type Pol I subunit Rpa43, or Rpa43^myc^. WCEs were prepared and subjected to IP experiments with anti-Myc antibody agarose followed by western blot analysis as described in the legend to Fig 2C. IgG on the right marks the positions of antibody chains in IPs. B) The CTR is hyperphosphorylated *in vivo*. ^FLAG^CTR in WCE of the diploid yeast strain W13490 was bound to anti-FLAG M2 antibody agarose and treated with increasing amounts (grey triangle) of λ-protein phosphatase (λ-PP). Protein samples were analyzed by western blot as described in the legend to Fig 2C. C) The CTR is differentially phosphorylated *in vivo*. Haploid yeast strain y3739 expressing ^FLAG^CTR was grown to exponential (exp), or to stationary phase (stat). WCE was prepared and subjected to western blot analysis with anti-FLAG antibody (αFLAG) as described in the legend to Fig 2C. Ponceau S stain of the membrane, and an image visualizing chemiluminescence signals upon immuno-detection (αFLAG) are shown. D) Recombinant CTR expressed in insect cells is hyperphosphorylated. Net1 CTR^TAP^ expressed in insect cells was bound to IgG-magnetic beads. Net1 CTR^TAP^ bound to the beads was either mock treated (-) or incubated with λ-PP (+). CTR^CBP^ fusion proteins were released by TEV protease cleavage and analyzed by SDS-PAGE and Coomassie staining. A photograph of the gel is shown. The size of molecular weight markers in kDa is indicated on the left. Positions of the tagged proteins as well as TEV protease are indicated on the right. E) CTR-dependent stimulation of Pol I transcription *in vitro* is decreased after λ-PP treatment. *In vitro* transcription reactions were carried out using Pol I purified from yeast, recombinant CF and Rrn3 purified from E. coli in the absence (-) or presence of increasing amounts (grey triangle) of CTR^CBP^, either mock treated or incubated with λ-PP as described in (D). Radiolabeled RNA was isolated and separated by 6% acrylamide-urea gel electrophoresis. An autoradiograph is shown. The position of the specific transcript is indicated on the right.

**Fig 8:**
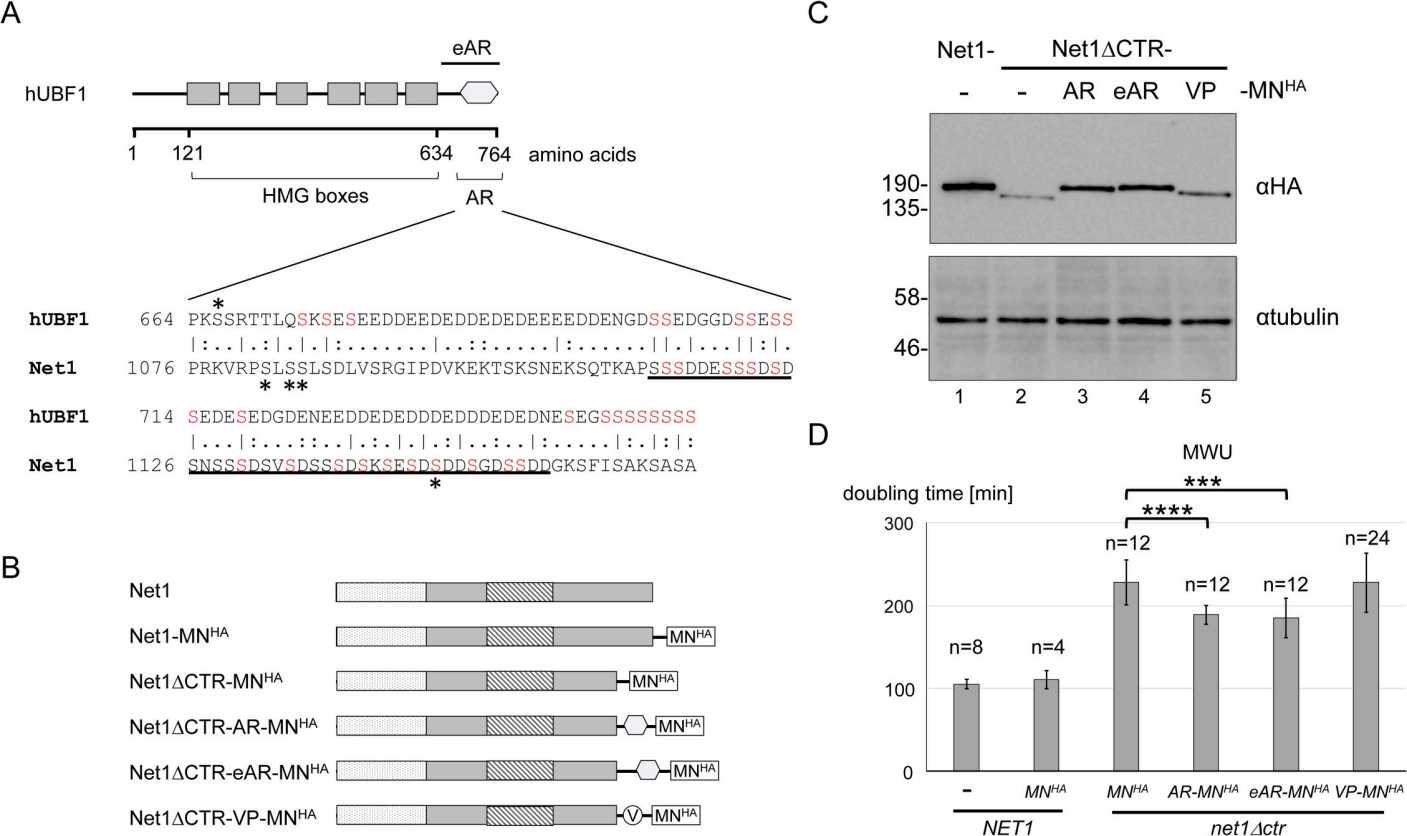
The acidic region of human UBF1 shares conserved features with the CTR and can partially rescue the growth defect of *net1Δctr* strains. (A) Local pairwise sequence alignment of human UBF1 (hUBF1) with Net1. Top, schematic representation of hUBF1 protein, depicting amino acid positions of full-length hUBF1, HMG boxes (grey rectangles), acidic tail region (AR, hexagon in light grey), and extended acidic tail region (eAR, black bar on the top). Bottom, sequence alignment of hUBF1 with Net1 using the “matcher” program EMBL-EBI Job Dispatcher framework (https://www.ebi.ac.uk/Tools/psa/emboss_matcher/). Identical (|), and similar (:) amino acid residues are indicated. Amino acid numbering of the full-length hUBF1 and yeast Net1 are indicated on the left, asterisks mark serine residues reported to be targets of phosphorylation (https://www.phosphosite.org/; https://www.yeastgenome.org), predicted CK2 phosphorylation sites are printed in red (http://www.cbs.dtu.dk/services/NetPhos/). A serine/aspartate rich amino acid stretch within the CTR is underlined. B-D) Diploid yeast strains were sporulated yielding haploid progenies expressing either wild-type Net1 or different Net1-MN^HA^ fusion proteins in which the CTR was either conserved (Net1-MN^HA^) deleted (Net1ΔCTR-MN^HA^), or replaced by the AR (Net1ΔCTR-AR-MN^HA^), or eAR regions (Net1ΔCTR-eAR-MN^HA^), or a synthetic, minimal transactivation domain of the viral VP16 protein (Net1ΔCTR-VP-MN^HA^). B) Schematic representation of Net1-MN^HA^ fusion proteins expressed in the respective yeast strains. Net1 domains are depicted as described in the legend to Fig 1B, UBF1 domains are depicted as described in (A). The minimal transactivation domain of VP16 is depicted as a circle. C) WCE of overnight cultures of haploid progenies (y4065, y4067, y4069, y4071, y4073), expressing the indicated Net1-MN^HA^ fusion proteins were subjected to western blot analysis as described in the legend to Fig 2C, using anti-HA antibody (αHA, top panel), or a tubulin antibody (αtubulin, bottom panel). Images visualizing chemiluminescence on the membranes are shown. D) Growth analyses in liquid culture were performed with the indicated haploid derivatives of yeast strains (y4038, y4039, y4040, y4041, y4042, y4043, y4044, y4045, y4046) as described in the legend to Fig 2B. Differences in doubling times of Net1ΔCTR-AR-MN^HA^ and Net1ΔCTR-eAR-MN^HA^ strain relative to the Net1ΔCTR-MN^HA^ expressing strain were tested using the nonparametric Mann-Whitney U test; ***P<0.001, ****P<0.0001.

Previous work showed co-purification of Net1 with Pol I subunits [42,46]. Here, it was tested if the CTR of Net1 could associate with Pol I. Immuno-precipitation experiments using whole-cell extracts from a strain expressing Rpa43^myc^ and ^FLAG^CTR with an anti-Myc antibody showed enrichment of the Myc-tagged bait protein and co-precipitation of a minor sub-population of ^FLAG^CTR in western blot analysis (Fig 7A, lanes 1 and 3). As expected, ^FLAG^CTR was not precipitated from whole-cell extracts of strains expressing the untagged Rpa43 protein (Fig 7A, lanes 2 and 4). ^FLAG^CTR in whole-cell extracts migrated as a diffuse band around 30 kDa in SDS-PAGE, despite an estimated molecular weight of 18 kDa. Net1 is known to be phosphorylated and several phosphorylation sites have been identified within the CTR in proteomic studies (Fig 8A, asterisks mark described phosphorylation sites) [32,47–50]. To investigate if the aberrant migration behavior of the CTR could be explained by phosphorylation, ^FLAG^CTR was enriched from whole-cell extracts using an anti-FLAG antibody matrix. The immobilized protein was incubated in the absence or presence of different concentrations of lambda protein phosphatase (λ PP). Western blot analysis revealed that the incubation with λ PP substantially increased the electrophoretic mobility of ^FLAG^CTR (Fig 7B, lanes 3–5; S4B Fig, full membrane) suggesting that the CTR is phosphorylated *in vivo*.

### Changes in post-translational covalent modifications of the CTR correlate with alterations in Pol I transcription of 35S rRNA genes *in vivo*

To investigate if the post-translational modification state of the CTR was subject to changes *in vivo*, whole-cell extracts were prepared from exponentially growing and stationary cells expressing ^FLAG^CTR. Proteins were separated by SDS-PAGE and analyzed in a western blot. ^FLAG^CTR in extracts from stationary cells had a higher mobility in SDS-PAGE than ^FLAG^CTR in extracts from exponentially growing cells, suggesting alterations in the modification state (Fig 7C, αFLAG, compare “exp” with “stat”; S4C Fig, full membrane). Ponceau-red staining of the immunoblot membrane indicated that the overall mobility of cellular proteins was not altered in the different extracts (Fig 7C, Ponceau; S4C Fig). Ribosome biogenesis and Pol I transcription are downregulated when yeast cells grow to stationary phase [51,52]. Downregulation of Pol I transcription correlates with a decreased psoralen accessibility of the 35S rRNA gene region transcribed by Pol I [53–55]. As a control for downregulation of Pol I transcription in the above experiment, we subjected cells from the same cultures used for protein extraction to psoralen crosslinking analysis. In agreement with the expected alterations in Pol I transcription, psoralen accessibility of 35S rRNA genes was high in exponentially growing cells and negligible in stationary phase cells (S4D Fig, compare lanes 4 and 5).

### λ protein phosphatase treatment decreases CTR-dependent stimulation of promoter-dependent Pol I transcription *in vitro*

Recombinant full-length Net1 has been shown to stimulate promoter-dependent Pol I transcription *in vitro* [42]. We tested if the CTR could be sufficient to promote Pol I stimulation in a reconstituted system. The CTR was expressed in insect cells with a C-terminal tandem affinity purification (TAP) tag (CTR^TAP^) bound to IgG-sepharose and released as a calmodulin binding peptide (CBP) fusion protein (CTR^CBP^) by cleavage with tobacco etch virus (TEV) protease (S4E Fig) [56]. As observed for the CTR expressed in exponentially growing yeast (Fig 7A–7C), purified recombinant CTR^CBP^ with a predicted size of 20 kDa migrated with an apparent molecular weight of 32 kDa in SDS-PAGE (Fig 7D; S4F Fig, lane1; S4E Fig, lane 3). λ PP treatment prior to TEV-mediated release from IgG-Sepharose significantly increased the mobility of the recombinant protein in SDS-PAGE when compared with the mobility of the mock-treated CTR^CBP^, indicating that the protein was phosphorylated (Fig 7D compare lane 1 with lane 2; S4F Fig, full gel). λ PP-treated and mock-treated CTR^CBP^ were tested in promoter-dependent Pol I transcription in a minimal *in vitro* system consisting of Pol I purified from yeast and of recombinant Rrn3 and CF purified from *E*. *coli* [57]. The mock treated CTR^CBP^ could stimulate promoter-dependent Pol I transcription in a concentration-dependent manner up to 6-fold, whereas the λ PP treated CTR^CBP^ still led to a 2 to 3-fold stimulation (Fig 7E, compare lanes 1 and 2, with lanes 3–6 and lanes 7–10). Additionally, recombinant full-length Net1^TAP^ and Net1ΔCTR^TAP^fusion proteins were purified from insect cells (S4G Fig) and tested in parallel with another preparation of CTR^CBP^ protein with and without λ PP treatment. The recombinant Net1^CBP^ was the strongest activator of Pol I transcription, whereas Net1ΔCTR^CBP^ still stimulated Pol I transcription to a similar extent as the purified CTR^CBP^ (S4H Fig). Thus, it is possible that Net1ΔCTR still partially supported Pol I transcription *in vivo*. In agreement with such hypothesis, residual Net1ΔCTR-MN^HA^-mediated cleavage at the 35S rDNA promoter could still be observed upon ChEC (Fig 4A, lanes 5–8; S3D Fig, upper panel, lanes 5–8). In contrast, ChEC analyses performed with cells from a Net1(1–341)-MN^HA^ expressing strain did not lead to significant cuts within the 35S rDNA promoter (S3D Fig, upper panel, lanes 9–12). Net1(1–341)-MN^HA^ mediated cleavage at the RFB region, instead, was comparable–although not identical—to cleavage mediated by Net1-MN^HA^ or by Net1ΔCTR-MN^HA^ (S3D Fig, lower panels).

### The acidic region of human UBF1 can in part functionally substitute for the CTR in the context of the Net1 protein

Whereas the full-length Net1 is not conserved in higher eukaryotes, local sequence alignment using the tool “matcher” of the EMBOSS platform revealed 25% identical and 43.5% similar residues within the acidic tail region (AR) of human UBF1 Pol I transcription factor (Fig 8A). An acidic serine/aspartate-rich sequence within the CTR showed the highest similarity with the UBF1 AR (Fig 8A, underlined amino acids). Assuming, that the CTR is hyperphosphorylated within this sequence, the similarity between the two regions might be even greater. To test if the UBF1 AR could functionally substitute for the Net1 CTR, yeast strains were created expressing different derivatives of Net1 in fusion with MN^HA^ (Fig 8B). Full-length Net1, Net1ΔCTR, or Net1 variants in which the CTR was replaced by the AR of human UBF1, an N-terminally extended AR (eAR), or a triplicated synthetic minimal VP16 activation domain (VP), respectively were analyzed. The VP16 activation domain triggers high-level Pol II-dependent transcription when recruited to gene promoters by a specific DNA binding domain in higher eukaryotes [58]. The expression of the respective MN^HA^-fusion proteins in the above strains was verified by western blot analysis (Fig 8C; S5A Fig, full membrane). Six independently obtained clones of each of these strains were subjected to growth analyses together with clones of a strain expressing an untagged Net1 protein as a reference control (Fig 8D; S1 Dataset). Whereas all the strains expressing Net1 variants lacking the CTR grew slower than the Net1-MN^HA^ or Net1-expressing strains, Net1ΔCTR-AR-MN^HA^ and Net1ΔCTR-eAR-MN^HA^-expressing strains grew slightly but statistically significantly faster than Net1ΔCTR-MN^HA^-expressing strains. This indicated that the Net1-CTR and the UBF1-AR regions share a conserved function in promoting growth. In contrast, expression of Net1ΔCTR-VP-MN^HA^ did not significantly rescue the *net1*Δ*ctr* growth phenotype in those experiments (Fig 8D).

## Discussion

In this study we could attribute the Pol I stimulating function of the multi-functional yeast Net1 protein to its C-terminal 138 amino acids (CTR). In good accordance with the presumed role of Pol I transcription in promoting cell proliferation, the CTR supported wild-type growth (Figs 2A, 2B, 3A and 3B; S1 Dataset). The CTR was further required for normal cellular morphology and localization of the nucleolar protein Nop56 (Figs 2G and 3D, S2A and S2B Fig). It is likely that the observed nucleolar de-localization of Nop56 in *net1Δctr* strains can be explained by impaired Pol I transcription. This would be in agreement with earlier observations showing that rRNA gene transcription by Pol I is an important determinant for proper localization of nucleolar components [59–61].

The nucleolar localization of Net1 correlates with its reported interaction with two different regions within the rDNA [37]. The CTR was dispensable for nucleolar localization of Net1 and for recruitment of Net1 and Cdc14 to the RFB element at the 3’-end of the 35S rRNA gene (Figs 2G and 4; S3D Fig). The latter observations are in good agreement with the observation that only the N-terminal 341 amino acids of Net1 are needed for its interaction with Fob1 [62], which in turn is needed for Net1 interaction with the RFB [37]. In contrast, the CTR was required for Net1 and Cdc14 association with the 35S rDNA promoter (Fig 4; S3D Fig). Secondary structure prediction did not provide evidence for the presence of a DNA binding domain within the CTR, but rather suggested that this protein region might be intrinsically disordered (S5B Fig). Thus, it is more likely that the CTR interacts with the promoter region in context of the DNA-bound protein (RNA) scaffold. Interestingly, interaction of Net1 with the 35S rDNA promoter was impaired in a *uaf30*Δ strain in which Pol I PIC formation is strongly affected [41], indicating that Net1 may interact with PIC components. Candidate interaction partners could be the UAF subunits Rrn5 and Rrn10, since they were found in a Cdc14 interactome study in which the entire RENT complex–including Net1—was purified [63]. Additionally, co-purification of many Pol I subunits with full-length Net1 was reported [37,42,64], and here, co-purification of the CTR with the Pol I subunit Rpa43 was shown (Fig 7A). A Pol I-related function of the CTR was further supported by the observation that the Pol I subunit Rpa190 in fusion with the CTR could suppress slow growth of *net1Δctr* strains (Fig 6C and 6D). Net1 interaction with the 5’ end of the 35S rDNA is not restricted to the promoter region but extends into the external transcribed spacer 1 as revealed in ChEC and ChIP experiments (Fig 4A; S3D Fig) [37,41]. Such a trailing into the Pol I transcribed 35S rDNA resembles the association observed for the Pol I transcription factor Rrn3 [65]. Thus, the CTR might interact with promoter-bound factors and assist efficient PIC assembly, and possibly additional downstream events of the Pol I transcription process.

The amino acid sequence analysis of the CTR revealed similarity with the AR of the human Pol I transcription factor UBF1 (Fig 8A). However, it should be noted that the CTR amino acid sequence is of low complexity, mainly composed out of aspartate and serine residues. Additionally, we used the HHPred module from the free HH-suite software package [66,67] to detect homologous domains in other proteins which identified a very similar (small) set of RNA-binding proteins for both CTR and AR (not shown). In our experiments, the AR of human UBF1 could at least partially substitute for the growth-supporting function of the CTR in the context of Net1 (Fig 8D; S1 Dataset). However, the observed effects on growth were small. Accordingly, Net1-(e)AR-MN^HA^ fusion proteins could not significantly restore cleavage at the 35S rDNA promoter in ChEC experiments (S5C Fig). Several studies have provided evidence that the carboxy-terminus of UBF1 is important to support efficient Pol I transcription, probably by stabilizing the UBF1-SL1 complex at the rDNA promoter [68–70]. Interestingly, the AR of UBF1 is also required for proper nucleolar localization of UBF1 and preferentially localizes to the nucleolus when expressed in *trans* [71,72], alike observations with the CTR of Net1 (Fig 3C and 3D; S2 Fig). Phosphorylation of specific residues within the acidic tail have been suggested to increase UBF-SL1 interaction [38,39,73–75], and UBF phosphorylation correlates well with active rRNA gene transcription *in vivo* [70,73,76–81]. Additionally, de-phosphorylation of UBF1 reduces its potential to stimulate promoter-dependent Pol I transcription *in vitro* [70,80]. Interestingly, a growth-dependent alteration in the post translational modification state could also be observed for the CTR, correlating with changes in Pol I transcription (Fig 7C; S4C and S4D Fig). Furthermore, de-phosphorylation decreased CTR-mediated stimulation of Pol I transcription *in vitro* (Fig 7E; S4H Fig). As a next step, it will be interesting to identify enzymes and pathways regulating the differential post-translational modification of the CTR. Two Cdc28 (Cdk1 in human) target sites reside within the CTR [32,48], and many residues within the serine-rich region are predicted to be potential substrates for casein kinase 2 (CK2; Fig 8A, predicted CK2 phosphorylation sites in bold [82,83]). Noteworthy, CK2 activity might have a function in the regulation of Pol I transcription [38,84,85]. The possibility that the CTR of Net1 functions in part similarly to the AR in UBF1 could point to further conserved mechanisms in Pol I transcription. As outlined in the introduction, the yeast HMG-box protein Hmo1 might also share functions with HMG-boxes 1 and 2 of UBF1 [17]. This could indicate that similar functional domains act in yeast and higher eukaryotes to support Pol I transcription but are organized in different polypeptides.

Recent advances in structure determination of the Pol I transcription machinery have yielded great insights in the architecture of individual components as well as in the assembly of a minimal PIC (reviewed in [86]). In the future, analyses using defined *in vitro* systems will further deduce structure-function relationships important to eventually understand the mechanism driving Pol I transcription. For faithful reconstitution of this process, it will be necessary to identify all of the participating factors *in vivo*. In this study, we identified another, likely conserved, component of the yeast Pol I transcription machinery.

## Materials and methods

### Oligonucleotides, plasmids and yeast strains

Unless noted otherwise, standard techniques were used for cloning of plasmids, and transformation of yeast cells [87–89]. Information about oligonucleotides, plasmids, and yeast strains used in this study can be found in S1–S3 Tables, including details on plasmid and strain construction. Plasmid sequences are available upon request. For the individual experiments in this study, yeast cells were grown at the indicated temperatures in either YPD (2% w/v) peptone, 1% (w/v) yeast extract, 2% glucose), YPAD (YPD including 100mg/l adenine), or XYD medium (YPD including 100mg/l adenine, 200mg/l tryptophan, and 10mM KH_2_PO_4_).

### Growth analyses

#### Spot assays

Cultures were grown at 25°C in XYD media overnight (*NET1*) or for 3 days (*net1*-mutants), respectively. Cells were collected by centrifugation and resuspended in sterile water to a final OD_600_ of 1. 10-fold serial dilutions of cell suspensions were spotted onto XYD agar plates and incubated at 25°C for several days. For experiments shown in Fig 6D cultures were grown for two days at 30°C in YPAD and spotted on YPAD plates. Photographs were taken at different times during the incubation to document growth.

#### Growth in liquid culture

Cultures were grown in YP(A)D at 30°C overnight. Cells were sedimented, washed with sterile water and inoculated into 200μl YPD in a sterile 96 well culture plate to a starting OD_600_ of 0.02. Incubation was carried out at 30°C under occasional shaking using a TECAN infinite 500 or 200Pro plate reader system (TECAN). The OD_600/613_ was determined every 15 min. Doubling time (τ) was calculated for the exponential growth phase by the formula τ = (t_2_-t_1_)/(log(2)/log(OD_t2_/OD_t1_) for more than 17 different time intervals. Mean doubling times and standard deviation errors for most of the strains used in this study can be found in S3 Table. All raw data of the TECAN measurements including growth diagrams shown in Figs 2B, 3B, 6D and 8D can be found in the S1 Dataset.

#### Statistics

Statistical analyses were performed with GraphPad Prism 5.04. Differences between groups were tested using the nonparametric two-sided Mann-Whitney U (MWU) test. *P*-values <0.05 were considered statistically significant.

### Live cell fluorescence microscopy

Exponentially growing cells were harvested by centrifugation, transferred to glass slides and covered with an agarose slice (supplied with yeast nitrogen base, amino acids, and 2% glucose). Cells were observed at 20–22°C on an Axio Observer Z.1 confocal spinning disk (CSU-X1) microscope (Yokogawa; Carl Zeiss) using a Plan Apochromat 63×/1.40 oil differential interference contrast (DIC) M27 lens. Ten Z-stack images with an optical section spacing of 0.5 μm were acquired with a shutter speed of 200 ms using an AxioCam MRm camera (Carl Zeiss). Microscopy data was processed using ImageJ (National Institutes of Health, Bethesda, MD). Z-stacks of the region of interest (fluorescence channels) were projected using maximum-intensity projection. A single plane (DIC channel) was duplicated to visualize cell morphology.

### Chromatin Endogenous Cleavage (ChEC), and Chromatin Immuno-Precipitation (ChIP)

For ChEC and ChIP experiments yeast strains expressing different genes as fusion proteins with a C-terminal MN followed by a triple HA-tag from their endogenous genomic location were grown in YP(A)D at 30°C to a final OD_600_ of 0.5. ChEC analyses were performed as previously described [15]. All DNA samples were digested with XcmI prior to agarose gel electrophoresis and Southern blot analysis (see below). ChIP experiments were performed as described and analyzed by quantitative PCR [41]. Data was collected with a Rotor-Gene Q system (Qiagen). The cycle threshold values and amplification efficiency (E) for a defined primer pair for all input and ChIP samples of an experimental dataset were determined with the Rotor-Gene 6000 software (Qiagen) using the comparative quantification method. The cycle threshold (CT) value correlates with the Rotor-Gene software “take-off” value (tov). For each primer pair the efficiency (E) is calculated as an average from the fluorescence measurements for each individual sample. The percentage of co-precipitated DNA was determined by the formula %ChIP = ((1+E)^tov(ChIP)^/(1+E)^tov(input)^)*100. Average and standard deviation errors for ChIP are derived from three independent ChIP experiments, each analyzed in triplicate qPCRs. The original qPCR data, including CT, “take off” and efficiency values, melting curve analysis, and mathematical operations are added as S2 Dataset.

### Gel electrophoresis and blot analysis

SDS–PAGE and Western blot analysis was performed according to standard procedures [90,91]. Gels were either stained with Coomassie blue for detection of proteins or transferred to nitro cellulose (GE Healthcare) or PVDF membranes (Immobilon P, Roth) for subsequent immuno-detection. Transfer to membranes was verified by staining with Ponceau S. S4 Table contains a complete list of antibodies used for detection. Secondary antibodies coupled to IRDye 800 were detected with an LI-COR Odyssey Infrared Imaging System (LI-COR Bioscience). Secondary antibodies coupled to horse reddish peroxidase (HRPO) were visualized using BM Chemiluminescence Western Blotting Substrate (POD, Roche), and a LAS-3000 Chemiluminescence Imager.

Agarose gel electrophoresis and Southern blot analysis were performed as described [88], with the exception that transfer of nucleic acids onto nylon membranes (Positive Membrane, Qbiogen) by capillary transfer was performed with 1 M ammonium acetate [92]. S5 Table contains a list of templates used for the synthesis of radiolabeled hybridization probes generated using the RadPrime DNA labeling system (GE Healthcare).

### Protein extraction, immuno-precipitation (IP) and λ-phosphatase treatment

For preparation of native protein extracts yeast cells were grown overnight in XYD media at 25°C to an OD_600_ of 0.8. For preparation of yeast whole-cell extracts (WCE), cell equivalents corresponding to 10–20 OD_600_ were harvested by centrifugation (805 rcf, 2 min) and washed once with ice-cold water. Lysis buffer (150 mM NaCl (for IPs with Net1^FLAG^-fragments) or 350 mM KCl (IPs with Rpa43^myc^), 50 mM Tris-HCl pH 7.5, 50 mM NaF, 5 mM EDTA, 0.1% IGEPAL CA-630, 60 mM β-glycerol phosphate) was added to yield a final volume of 150 μl cell suspension. After addition of 200μl of glass beads cells were lysed by shaking the mixture for 5 min at 4°C in a mixer mill (Retsch). After removal of cell debris by two consecutive centrifugation steps of 10 min and 15 min (16000 rcf, 4°C), equal volumes of supernatant and 2× SDS-sample buffer were mixed and incubated for 10 min at 100°C.

For immuno-precipitation of Myc-epitope tagged RNA polymerase I subunits, WCEs (adjusted to a total volume of 450μl) were incubated with anti-Myc antibodies (clone 9E10) for 2h at 4°C. Thereafter, 50 μl of protein A–agarose slurry (Santa Cruz Biotechnology Inc.) were added, and the mixtures were incubated on a rotator for 2 h at 4°C. For immuno-precipitation of FLAG-tagged Net1-fragments, WCEs (adjusted to a total volume of 450 μl) were incubated with 45μl of α-FLAG-beads (Sigma-Aldrich) on a rotator for 3 h at 4°C. Beads were collected by centrifugation and washed three times with 500 μl lysis buffer. After the last washing step, beads were resuspended in 1x SDS sample buffer and incubated for 10 min at 100°C.

After immuno-precipitation of FLAG-tagged CTR as described above, beads were collected by centrifugation and washed twice with 1ml of lysis buffer (without β-glycerol phosphate) and once with λ-phosphatase buffer (50 mM HEPES-KOH pH 7.5, 5 mM DTT, 2 mM MnCl_2_). Beads were split in different samples and treated in the absence (control), or presence of 200, 400, and 2000 units of λ-phosphatase (New England Biolabs) in a total volume of 50μl λ-phosphatase buffer, and samples were incubated for 1 h at 37°C. Beads were collected by centrifugation and washed twice with 1 ml λ-phosphatase buffer. After the last washing step, beads were resuspended in 1x SDS-sample buffer and incubated for 10 min at 100°C.

All samples were analyzed by western blot as described above. The data presented in Figs 2C, 7A and 7B are representative for at least three independent experiments.

### Yeast cell growth to stationary phase with subsequent protein and trimethyl psoralen crosslinking analysis

Single colonies of yeast strains were used to inoculate 3 ml of YPAD which were incubated for 2 days at 30°C under shaking. This culture was used to inoculate YPAD media to an OD_600_ not higher than 0.1. Samples for denaturing protein extraction or trimethyl psoralen crosslinking analyses were withdrawn when the culture reached an OD600 of 0.5 (exponential phase) or 144 hours after this time point (stationary phase). For protein analysis cell equivalents of 0.5–3 OD_600_, were subjected to protein extraction as described elsewhere [93]. For trimethyl-psoralen crosslinking analysis, cell equivalents of 25 OD_600_ were treated as previously described [15]. All DNA samples were digested with EcoRI prior to agarose gel electrophoresis and Southern blot analysis (see above).

### Purification of recombinant proteins and λ-phosphatase treatment

Purification of 6x histidine-tagged Rrn3, or 6x histidine-tagged core factor from *E*. *coli*, as well as TAP-tagged RNA Pol I from yeast have been described previously [57]. Recombinant expression of different Net1-TAP fusion proteins in baculovirus infected *S*. *frugipedia* SF21 cells was achieved according to a protocol described in [94–96]. For TAP-tag mediated purification, 50x10^6^ cells from a large scale infection were suspended in 30 ml TAP-lysis buffer (50 mM HEPES-KOH pH 7.5, 2.5 mM Mg-acetate, 0.001% (w/v) Tween, 0.2 M KCl, 5 mM 2-mercaptoethanol, 1 mM PMSF and 2 mM benzamidine) and whole-cell lysates were prepared as previously described [96]. To purify TAP tagged proteins from 30ml of extract were added to 75mg of magnetic beads (BcMag, Bioclone Inc.) coupled to rabbit IgGs (Sigma) prepared as described [97]. Further purification followed the protocol for TAP-tagged RNA Pol I from yeast [57].

Dephosphorylation of recombinant CTR-TAP fusion protein was performed similarly to the dephosphorylation of the FLAG-tagged CTR purified from yeast (see above). In brief, after binding TAP-tagged CTR to IgG-coated magnetic beads (see above), the affinity matrix was washed 3x with 1 ml TAP-lysis buffer, and 3x with 1 ml TAP-lysis buffer lacking PMSF and benzamidine. The magnetic beads were equilibrated with 1 ml λ-phosphatase buffer and split into two aliquots. Beads in each aliquot were suspended in a total volume of 50 μl λ-phosphatase buffer in the absence (mock control) or presence of 2000 u λ-phosphatase and incubated in a thermomixer (Eppendorf) for 1h at 30°C under shaking. The supernatant was discarded, and the beads were washed 3x with 1 ml TAP-lysis buffer lacking PMSF and benzamidine. Proteins bound to the beads were eluted by TEV cleavage as previously described [57]. This experiment has been repeated three times and a representative experiment is shown in Fig 7D.

### Promoter-dependent RNA polymerase I *in vitro* transcription

*In vitro* transcription analysis was performed essentially as previously described [57], with minor changes. To allow formation of the initiation-competent Rrn3-Pol I complex, 5 nM RNA Pol I and 70 nM Rrn3 were incubated at 4°C for 60 min. The reaction was complemented with 10 nM CF, and 5 nM template DNA containing the 35S rDNA promoter. Additionally, potassium acetate was added to yield a constant salt concentration of 200 mM in the final transcription reaction. In some reactions affinity purified Net1 derivatives were included (5–20 nM for full-length Net1, and Net1ΔCTR, and 20–100 nM for CTR or dephosphorylated CTR). The reaction was eventually adjusted to 25μl containing 20 mM HEPES-KOH pH 7.8, 10 mM MgCl_2_, 5 mM EGTA, 0.05 mM EDTA, 2.5 mM DTT, 0.2 mM ATP, 0.2 mM UTP, 0.2 mM GTP, 0.01 mM CTP including 1.25 μM α-^32^P-CTP. Samples were incubated for 30 min at 24°C, before the reaction was stopped and processed as reported [57]. The data presented in Fig 7E and S4H Fig are representative for at least three independent experiments.

## Supporting information

S1 Fig Yeast strains expressing Net1 protein with a C-terminal deletion are affected in growth but produce a protein which still interacts with Cdc14 and Sir2. A) WCEs of diploid yeast strains (W13533, W13534, W13535, W13536, W13537, W13538, W13539, W11979) (lanes 1–7), or the respective haploid progenies (lanes 8–14), expressing the indicated GFP fusion proteins, were prepared and subjected to western blot analysis with anti-GFP antibody (αGFP, upper panel) and anti-tubulin antibody (αtubulin, lower panel) as described in the legend to Fig 2C. B) Diploid yeast strains (W13533, W13534, W13535, W13536, W13537, W13538, W13539, W11979) were sporulated yielding haploid progenies carrying alleles for expression of the indicated GFP-fusion proteins. Cartoons of the Net1-GFP proteins are depicted on the right according to Fig 1B. Serial dilutions of cell suspensions of the haploid strains and a control strain K699 carrying a *NET1* wild-type allele were spotted on XYD plates and incubated at 25°C for 2d before a photograph was taken. C) Pictures of the full-size membranes shown in Fig 2C (see legend to this figure for more information). An asterisk on the right marks bands which are the result of cross-hybridization of antibodies with cellular proteins in WCEs and chains of antibody used for the IPs.
(TIF)

S2 Fig Expression of the CTR in *trans* rescues growth defects and Nop56 delocalization in *net1Δctr* strains. A-C) CTR expression in *trans* re-establishes wild-type cell morphology and Nop56 nucleolar localization in *net1(1–455)* strains and has a preferential nucleolar localization in *net1Δ* strains.
A,B) The diploid yeast strain W15406 was sporulated, yielding haploid progenies carrying a *net1(1–455)* allele, and expressing Nop56^mCherry^ in the absence (A) and presence of ^GFP^CTR (B). Haploid strains were subjected to live cell fluorescence microscopy as described in the Legend to Fig 2D. C) The diploid yeast strain W12509 was sporulated, yielding haploid progenies carrying a *net1*Δ allele, and expressing ^GFP^CTR. The haploid strain was subjected to live cell fluorescence microscopy as described in the Legend to Fig 2D.
D,E) ^FLAG^CTR expression suppresses the growth defect of *net1Δctr* strains and does not alter expression levels of Net1ΔCTR^FLAG^
Haploid yeast strains (y3058, y3068, y3250), carrying a *NET1* or a *net1Δctr* allele, and expressing Cdc14-MN^HA^ in the absence or presence of a chromosomally integrated expression cassette for ^FLAG^CTR were subjected to growth and western blot analyses. D) Growth analyses in liquid culture were performed as described in the legend to Fig 2B. E) WCEs were prepared and subjected to western blot analysis as described in the legend to Fig 2C, using anti-FLAG antibody (αFLAG, top panel), or anti-HA antibody (αHA, bottom panel). The positions of tagged proteins on the membrane, as well as of degradation products of Net1ΔCTR^FLAG^ are indicated on the right.
(TIF)

S3 Fig Association of RENT complex components with rDNA is impaired in *net1Δctr* strains. Haploid yeast strains were subjected to ChEC (A,C,D) and ChIP (B) analyses as described in the legend to Fig 4.
A-C) Association of Fob1 and Sir2 with rDNA is impaired in *net1Δctr* strains
A,B) ChEC and ChIP analyses with strains y952, and y3040, carrying a *NET1* or a *net1Δctr* allele, and expressing Fob1-MN^HA^. C) ChEC analyses with strains y1450, and y2966, carrying a *NET1* or a *net1Δctr* allele, and expressing Sir2-MN^HA^. Two asterisks label the position of a fragment which was dependent upon the addition of calcium to the crude nuclei. This fragment was unrelated to Sir2-MN^HA^ since it was also observed in strains not expressing any MN fusion protein (not shown).
D) C-terminal truncation of Net1 abolishes association with the 35S rDNA promoter and cannot be restored upon expression of ^FLAG^CTR
ChEC analyses with yeast strains (y3157; y3164; y3145), expressing Net1-MN^HA^, Net1ΔCTR-MN^HA^, or Net1(1–341)-MN^HA^ from the endogenous *NET1* locus, and ^FLAG^CTR from a chromosomally integrated cassette.
(TIF)

S4 Fig The CTR is differentially phosphorylated *in vivo* and stimulates promoter-dependent Pol I transcription *in vitro*. A-C) The CTR is differentially phosphorylated in exponentially growing and stationary yeast cells *in vivo* A-C) Fluorographs and Ponceau staining of the full-size membranes shown in Fig 6A–6C (see legend to these figures for more information). D) Haploid yeast strain y3739 expressing ^FLAG^CTR was cultured in YPD. Different samples were withdrawn at exponential (exp) and stationary phase (stat), and either used for protein extraction (Fig 6C; S4C Fig) or treated with formaldehyde for psoralen crosslinking analysis. Crude nuclear extracts were prepared from formaldehyde treated cells, which were subjected to the psoralen crosslinking procedure. DNA was isolated, digested with EcoRI, separated by native agarose gel and subjected to Southern blot analyses with probe “3.5kb rDNA”. Positions of rDNA fragments derived from Pol I transcribed “open” 35S rRNA genes and nucleosomal “closed” 35S rRNA genes are depicted on the right. An autoradiography of the Southern blot membrane is shown. Lanes 1 and 2 show the analysis of a control experiment with strain y3725, which did not express ^FLAG^CTR. Lane C included genomic DNA digested with EcoRI, isolated from a strain which was not subjected to psoralen crosslinking but mock treated in parallel with the other strains. The sizes of two DNA fragments in kb spanning either parts of the 18S or the 25S rDNA transcribed by Pol I and visualized by probe “3.5kb rDNA” are depicted on the left.
E-H) Recombinant Net1, Net1DCTR and CTR purified from insect cells stimulate promoter-dependent Pol I transcription *in vitro*, and CTR-mediated stimulation of *in vitro* transcription is reduced upon λ-protein phosphatase treatment
E,G) Purification of recombinant Net1^TAP^ variants from bacmid infected Sf9 cells was performed as described in the legend to Fig 6D. Proteins contained in samples of the whole-cell extract (WCE, 0.06% of total), the flow through (FT, 0.06%), elution 1 and 2 after cleavage with TEV protease (E1, E2, 20%), and beads after elution (B, 20%) were analyzed by SDS PAGE and Coomassie blue staining. Photographs of the gels are shown. Molecular weights and positions of marker proteins are indicated on the left. F) Photograph of the entire gel of the experiment described in the legend to Fig 6D. H) *In vitro* transcription reactions were carried out using a minimal system containing Pol I purified from yeast, recombinant CF and Rrn3 purified from E. coli in the absence (-) or presence of increasing amounts (grey triangle) of Net1^CBP^, Net1ΔCTR^CBP^, or CTR^CBP^, which was either mock treated or incubated with λ-PP as described in the legend to Fig 6E. Plasmid 435 was linearized with BstZ17I and was used as Pol I promoter carrying template DNA which should yield a 591bp run-off transcript. Radiolabeled RNA was isolated and separated by 6% acrylamide-urea gel electrophoresis. An autoradiograph is shown. The position the specific transcript is indicated on the right.
(TIF)

S5 Fig The acidic region of human UBF1 shares conserved features with the CTR and can partially rescue the growth defect of *net1Δctr* strains but does not significantly increase the interaction of the respective Net1Δctr fusion proteins with the 35S rDNA promoter. A) Chemiluminescence on the full-size membranes shown in Fig 7C. B) Secondary structure prediction by the Disorder Prediction Meta-Server (http://www-nmr.cabm.rutgers.edu/bioinformatics/disorder/) for yeast Net1 (amino acids (aa) 1–1189; GI: 1023942937) and hUBF1 (aa 1–764; GI: 7657671). The disorder consensus model was created using 6 public structure prediction tools. The Disorder consensus (1 = disordered, 0 = structured) is plotted against the amino acid sequence of full-length yeast Net1 (top), and full length hUBF1. Scaled cartoons of Net1 and hUBF1 are depicted below the respective graphs with symbols described in the legends to Fig 1B and Fig 7A, respectively. A red dotted rectangle frames the regions shown in the pairwise sequence alignment in Fig 7A. C) Haploid yeast strains (y4066; y4068; y4070; y4072; y4074) expressing the indicated MN^HA^ fusion proteins were subjected to ChEC analyses as described in Fig 4.
(TIF)

S1 Table Oligonucleotides used in this study. The respective oligonucleotide names/database numbers, sequences, as well as a short description are given.
(XLSX)

S2 Table Plasmids used in this study. The respective plasmid names/database number, details about plasmid construction, as well as a short description are given.
(XLSX)

S3 Table Yeast strains used in this study and parental strains used for strain construction. For each strain, the name/database number, relevant and complete genotype, parental strain, details about strain construction, doubling times (if available), and information about spores analyzed (if applicable) are given. The relevant figures in which the results of experiments with the respective yeast strains are shown are listed.
(XLSX)

S4 Table Antibodies used in this study. (XLSX)

S5 Table Templates used for Southern blot probe generation. (XLSX)

S1 Dataset Raw data of growth analyses using automated OD measurement by TECAN reader systems. Growth analysis of liquid cultures was performed in a TECAN reader system in 96-well culture plates at 30°C under occasional shaking. The optical density at 600/612nm of each culture was determined in 15 minute time intervals. The coordinates of the 96-well plate, strain names/database numbers, cultivation times and OD measurements are given. The OD was subsequently corrected for the blank, which was determined individually in each experiment. The OD or the logarithm of the OD were plotted against the culture time. OD values in the exponential growth phase were used to calculate doubling times presented in Figs 2B, 3B, 6D and 8D.
(XLSX)

S2 Dataset Raw data of quantitative PCR analysis performed in the RotorGene Q system. Representative ChIP data of three individual experiments. CT values for each sample are given. The fluorescence measurements plotted against the cycle number and melting curve analyses are shown as diagrams. Additionally, values for “take-off” and “amplification” which are used by the comparative quantitation module of the RotorGene software to compute the “comparative concentration” used to calculate the % IP values and standard deviation errors are shown.
(XLSX)

## References

[1] Warner JR. The economics of ribosome biosynthesis in yeast. Trends Biochem Sci. 1999 11;24(11):437–40. 10542411 10.1016/s0968-0004(99)01460-7

[2] Goodfellow SJ, Zomerdijk JCBM. Basic mechanisms in RNA polymerase I transcription of the ribosomal RNA genes. Subcell Biochem. 2013;61:211–36. 10.1007/978-94-007-4525-4_10 23150253 PMC3855190

[3] Moss T, Langlois F, Gagnon-Kugler T, Stefanovsky V. A housekeeper with power of attorney: the rRNA genes in ribosome biogenesis. Cell Mol Life Sci CMLS. 2007 1;64(1):29–49. 10.1007/s00018-006-6278-1 17171232 PMC11136161

[4] Boukhgalter B, Liu M, Guo A, Tripp M, Tran K, Huynh C, et al Characterization of a fission yeast subunit of an RNA polymerase I essential transcription initiation factor, SpRrn7h/TAF(I)68, that bridges yeast and mammals: association with SpRrn11h and the core ribosomal RNA gene promoter. Gene. 2002 5 29;291(1–2):187–201. 12095692 10.1016/s0378-1119(02)00597-8

[5] Keys DA, Vu L, Steffan JS, Dodd JA, Yamamoto RT, Nogi Y, et al RRN6 and RRN7 encode subunits of a multiprotein complex essential for the initiation of rDNA transcription by RNA polymerase I in Saccharomyces cerevisiae. Genes Dev. 1994 10 1;8(19):2349–62. 7958901 10.1101/gad.8.19.2349

[6] Knutson BA, Hahn S. Yeast Rrn7 and human TAF1B are TFIIB-related RNA polymerase I general transcription factors. Science. 2011 9 16;333(6049):1637–40. 10.1126/science.1207699 21921198 PMC3319074

[7] Lalo D, Steffan JS, Dodd JA, Nomura M. RRN11 encodes the third subunit of the complex containing Rrn6p and Rrn7p that is essential for the initiation of rDNA transcription by yeast RNA polymerase I. J Biol Chem. 1996 8 30;271(35):21062–7. 8702872 10.1074/jbc.271.35.21062

[8] Milkereit P, Tschochner H. A specialized form of RNA polymerase I, essential for initiation and growth-dependent regulation of rRNA synthesis, is disrupted during transcription. EMBO J. 1998 7 1;17(13):3692–703. 10.1093/emboj/17.13.3692 9649439 PMC1170705

[9] Miller G, Panov KI, Friedrich JK, Trinkle-Mulcahy L, Lamond AI, Zomerdijk JC. hRRN3 is essential in the SL1-mediated recruitment of RNA Polymerase I to rRNA gene promoters. EMBO J. 2001 3 15;20(6):1373–82. 10.1093/emboj/20.6.1373 11250903 PMC145519

[10] Steffan JS, Keys DA, Dodd JA, Nomura M. The role of TBP in rDNA transcription by RNA polymerase I in Saccharomyces cerevisiae: TBP is required for upstream activation factor-dependent recruitment of core factor. Genes Dev. 1996 10 15;10(20):2551–63. 8895657 10.1101/gad.10.20.2551

[11] Bell SP, Learned RM, Jantzen HM, Tjian R. Functional cooperativity between transcription factors UBF1 and SL1 mediates human ribosomal RNA synthesis. Science. 1988 9 2;241(4870):1192–7. 3413483 10.1126/science.3413483

[12] Bell SP, Jantzen HM, Tjian R. Assembly of alternative multiprotein complexes directs rRNA promoter selectivity. Genes Dev. 1990 6;4(6):943–54. 2384215 10.1101/gad.4.6.943

[13] Herdman C, Mars J-C, Stefanovsky VY, Tremblay MG, Sabourin-Felix M, Lindsay H, et al A unique enhancer boundary complex on the mouse ribosomal RNA genes persists after loss of Rrn3 or UBF and the inactivation of RNA polymerase I transcription. PLoS Genet. 2017 7;13(7):e1006899 10.1371/journal.pgen.1006899 28715449 PMC5536353

[14] Sanij E, Poortinga G, Sharkey K, Hung S, Holloway TP, Quin J, et al UBF levels determine the number of active ribosomal RNA genes in mammals. J Cell Biol. 2008 12 29;183(7):1259–74. 10.1083/jcb.200805146 19103806 PMC2606969

[15] Merz K, Hondele M, Goetze H, Gmelch K, Stoeckl U, Griesenbeck J. Actively transcribed rRNA genes in S. cerevisiae are organized in a specialized chromatin associated with the high-mobility group protein Hmo1 and are largely devoid of histone molecules. Genes Dev. 2008 5 1;22(9):1190–204. 10.1101/gad.466908 18451108 PMC2335315

[16] Wittner M, Hamperl S, Stöckl U, Seufert W, Tschochner H, Milkereit P, et al Establishment and maintenance of alternative chromatin states at a multicopy gene locus. Cell. 2011 5 13;145(4):543–54. 10.1016/j.cell.2011.03.051 21565613

[17] Albert B, Colleran C, Léger-Silvestre I, Berger AB, Dez C, Normand C, et al Structure-function analysis of Hmo1 unveils an ancestral organization of HMG-Box factors involved in ribosomal DNA transcription from yeast to human. Nucleic Acids Res. 2013 12;41(22):10135–49. 10.1093/nar/gkt770 24021628 PMC3905846

[18] Drygin D, Rice WG, Grummt I. The RNA polymerase I transcription machinery: an emerging target for the treatment of cancer. Annu Rev Pharmacol Toxicol. 2010;50:131–56. 10.1146/annurev.pharmtox.010909.105844 20055700

[19] Kusnadi EP, Hannan KM, Hicks RJ, Hannan RD, Pearson RB, Kang J. Regulation of rDNA transcription in response to growth factors, nutrients and energy. Gene. 2015 2 1;556(1):27–34. 10.1016/j.gene.2014.11.010 25447905

[20] de la Cruz J, Gómez-Herreros F, Rodríguez-Galán O, Begley V, de la Cruz Muñoz-Centeno M, Chávez S. Feedback regulation of ribosome assembly. Curr Genet. 2018 4;64(2):393–404. 10.1007/s00294-017-0764-x 29022131

[21] Clemente-Blanco A, Mayán-Santos M, Schneider DA, Machín F, Jarmuz A, Tschochner H, et al Cdc14 inhibits transcription by RNA polymerase I during anaphase. Nature. 2009 3 12;458(7235):219–22. 10.1038/nature07652 19158678 PMC4445138

[22] Voit R, Seiler J, Grummt I. Cooperative Action of Cdk1/cyclin B and SIRT1 Is Required for Mitotic Repression of rRNA Synthesis. PLoS Genet. 2015 5;11(5):e1005246 10.1371/journal.pgen.1005246 26023773 PMC4449194

[23] Muth V, Nadaud S, Grummt I, Voit R. Acetylation of TAF(I)68, a subunit of TIF-IB/SL1, activates RNA polymerase I transcription. EMBO J. 2001 3 15;20(6):1353–62. 10.1093/emboj/20.6.1353 11250901 PMC145524

[24] Bryk M, Banerjee M, Murphy M, Knudsen KE, Garfinkel DJ, Curcio MJ. Transcriptional silencing of Ty1 elements in the RDN1 locus of yeast. Genes Dev. 1997 1 15;11(2):255–69. 9009207 10.1101/gad.11.2.255

[25] Smith JS, Boeke JD. An unusual form of transcriptional silencing in yeast ribosomal DNA. Genes Dev. 1997 1 15;11(2):241–54. 9009206 10.1101/gad.11.2.241

[26] Shou W, Seol JH, Shevchenko A, Baskerville C, Moazed D, Chen ZW, et al Exit from mitosis is triggered by Tem1-dependent release of the protein phosphatase Cdc14 from nucleolar RENT complex. Cell. 1999 4 16;97(2):233–44. 10219244 10.1016/s0092-8674(00)80733-3

[27] Straight AF, Shou W, Dowd GJ, Turck CW, Deshaies RJ, Johnson AD, et al Net1, a Sir2-associated nucleolar protein required for rDNA silencing and nucleolar integrity. Cell. 1999 4 16;97(2):245–56. 10219245 10.1016/s0092-8674(00)80734-5

[28] Visintin R, Hwang ES, Amon A. Cfi1 prevents premature exit from mitosis by anchoring Cdc14 phosphatase in the nucleolus. Nature. 1999 4 29;398(6730):818–23. 10.1038/19775 10235265

[29] Cuperus G, Shafaatian R, Shore D. Locus specificity determinants in the multifunctional yeast silencing protein Sir2. EMBO J. 2000 6 1;19(11):2641–51. 10.1093/emboj/19.11.2641 10835361 PMC212746

[30] Traverso EE, Baskerville C, Liu Y, Shou W, James P, Deshaies RJ, et al Characterization of the Net1 cell cycle-dependent regulator of the Cdc14 phosphatase from budding yeast. J Biol Chem. 2001 6 15;276(24):21924–31. 10.1074/jbc.M011689200 11274204

[31] Hannig K. Net1—ein modular aufgebautes und multifunktionales Protein im Nukleolus der Hefe Saccharomyces cerevisiae [Internet] [phd]. 2016 [cited 2018 Dec 29]. Available from: https://epub.uni-regensburg.de/32428/

[32] Azzam R, Chen SL, Shou W, Mah AS, Alexandru G, Nasmyth K, et al Phosphorylation by cyclin B-Cdk underlies release of mitotic exit activator Cdc14 from the nucleolus. Science. 2004 7 23;305(5683):516–9. 10.1126/science.1099402 15273393

[33] Kuilman T, Maiolica A, Godfrey M, Scheidel N, Aebersold R, Uhlmann F. Identification of Cdk targets that control cytokinesis. EMBO J. 2015 1 2;34(1):81–96. 10.15252/embj.201488958 25371407 PMC4291482

[34] Shou W, Azzam R, Chen SL, Huddleston MJ, Baskerville C, Charbonneau H, et al Cdc5 influences phosphorylation of Net1 and disassembly of the RENT complex. BMC Mol Biol. 2002 4 17;3:3 10.1186/1471-2199-3-3 11960554 PMC113746

[35] Visintin R, Stegmeier F, Amon A. The role of the polo kinase Cdc5 in controlling Cdc14 localization. Mol Biol Cell. 2003 11;14(11):4486–98. 10.1091/mbc.E03-02-0095 14551257 PMC266767

[36] Yoshida S, Toh-e A. Budding yeast Cdc5 phosphorylates Net1 and assists Cdc14 release from the nucleolus. Biochem Biophys Res Commun. 2002 6 14;294(3):687–91. 10.1016/S0006-291X(02)00544-2 12056824

[37] Huang J, Moazed D. Association of the RENT complex with nontranscribed and coding regions of rDNA and a regional requirement for the replication fork block protein Fob1 in rDNA silencing. Genes Dev. 2003 9 1;17(17):2162–76. 10.1101/gad.1108403 12923057 PMC196457

[38] Lin C-Y, Navarro S, Reddy S, Comai L. CK2-mediated stimulation of Pol I transcription by stabilization of UBF-SL1 interaction. Nucleic Acids Res. 2006;34(17):4752–66. 10.1093/nar/gkl581 16971462 PMC1635259

[39] Tuan JC, Zhai W, Comai L. Recruitment of TATA-binding protein-TAFI complex SL1 to the human ribosomal DNA promoter is mediated by the carboxy-terminal activation domain of upstream binding factor (UBF) and is regulated by UBF phosphorylation. Mol Cell Biol. 1999 4;19(4):2872–9. 10082553 10.1128/mcb.19.4.2872 PMC84080

[40] Bairwa NK, Zzaman S, Mohanty BK, Bastia D. Replication Fork Arrest and rDNA Silencing Are Two Independent and Separable Functions of the Replication Terminator Protein Fob1 of Saccharomyces cerevisiae. J Biol Chem. 2010 4 23;285(17):12612–9. 10.1074/jbc.M109.082388 20179323 PMC2857089

[41] Goetze H, Wittner M, Hamperl S, Hondele M, Merz K, Stoeckl U, et al Alternative chromatin structures of the 35S rRNA genes in Saccharomyces cerevisiae provide a molecular basis for the selective recruitment of RNA polymerases I and II. Mol Cell Biol. 2010 4;30(8):2028–45. 10.1128/MCB.01512-09 20154141 PMC2849473

[42] Shou W, Sakamoto KM, Keener J, Morimoto KW, Traverso EE, Azzam R, et al Net1 stimulates RNA polymerase I transcription and regulates nucleolar structure independently of controlling mitotic exit. Mol Cell. 2001 7;8(1):45–55. 11511359 10.1016/s1097-2765(01)00291-x

[43] Schmid M, Durussel T, Laemmli UK. ChIC and ChEC; genomic mapping of chromatin proteins. Mol Cell. 2004 10 8;16(1):147–57. 10.1016/j.molcel.2004.09.007 15469830

[44] Wu C. The 5’ ends of Drosophila heat shock genes in chromatin are hypersensitive to DNase I. Nature. 1980 8 28;286(5776):854–60. 6774262 10.1038/286854a0

[45] Stegmeier F, Huang J, Rahal R, Zmolik J, Moazed D, Amon A. The replication fork block protein Fob1 functions as a negative regulator of the FEAR network. Curr Biol CB. 2004 3 23;14(6):467–80. 10.1016/j.cub.2004.03.009 15043811

[46] Huang J, Brito IL, Villén J, Gygi SP, Amon A, Moazed D. Inhibition of homologous recombination by a cohesin-associated clamp complex recruited to the rDNA recombination enhancer. Genes Dev. 2006 10 15;20(20):2887–901. 10.1101/gad.1472706 17043313 PMC1619942

[47] Albuquerque CP, Smolka MB, Payne SH, Bafna V, Eng J, Zhou H. A multidimensional chromatography technology for in-depth phosphoproteome analysis. Mol Cell Proteomics MCP. 2008 7;7(7):1389–96. 10.1074/mcp.M700468-MCP200 18407956 PMC2493382

[48] Holt LJ, Tuch BB, Villén J, Johnson AD, Gygi SP, Morgan DO. Global analysis of Cdk1 substrate phosphorylation sites provides insights into evolution. Science. 2009 9 25;325(5948):1682–6. 10.1126/science.1172867 19779198 PMC2813701

[49] Soulard A, Cremonesi A, Moes S, Schütz F, Jenö P, Hall MN. The rapamycin-sensitive phosphoproteome reveals that TOR controls protein kinase A toward some but not all substrates. Mol Biol Cell. 2010 10 1;21(19):3475–86. 10.1091/mbc.E10-03-0182 20702584 PMC2947482

[50] Swaney DL, Beltrao P, Starita L, Guo A, Rush J, Fields S, et al Global analysis of phosphorylation and ubiquitylation cross-talk in protein degradation. Nat Methods. 2013 7;10(7):676–82. 10.1038/nmeth.2519 23749301 PMC3868471

[51] Claypool JA, French SL, Johzuka K, Eliason K, Vu L, Dodd JA, et al Tor pathway regulates Rrn3p-dependent recruitment of yeast RNA polymerase I to the promoter but does not participate in alteration of the number of active genes. Mol Biol Cell. 2004 2;15(2):946–56. 10.1091/mbc.E03-08-0594 14595104 PMC329406

[52] Ju Q, Warner JR. Ribosome synthesis during the growth cycle of Saccharomyces cerevisiae. Yeast Chichester Engl. 1994 2;10(2):151–7. 10.1002/yea.320100203 8203157

[53] Dammann R, Lucchini R, Koller T, Sogo JM. Chromatin structures and transcription of rDNA in yeast Saccharomyces cerevisiae. Nucleic Acids Res. 1993 5 25;21(10):2331–8. 8506130 10.1093/nar/21.10.2331 PMC309528

[54] Fahy D, Conconi A, Smerdon MJ. Rapid changes in transcription and chromatin structure of ribosomal genes in yeast during growth phase transitions. Exp Cell Res. 2005 5 1;305(2):365–73. 10.1016/j.yexcr.2005.01.016 15817161

[55] Sandmeier JJ, French S, Osheim Y, Cheung WL, Gallo CM, Beyer AL, et al RPD3 is required for the inactivation of yeast ribosomal DNA genes in stationary phase. EMBO J. 2002 9 16;21(18):4959–68. 10.1093/emboj/cdf498 12234935 PMC126294

[56] Rigaut G, Shevchenko A, Rutz B, Wilm M, Mann M, Séraphin B. A generic protein purification method for protein complex characterization and proteome exploration. Nat Biotechnol. 1999 10;17(10):1030–2. 10.1038/13732 10504710

[57] Pilsl M, Crucifix C, Papai G, Krupp F, Steinbauer R, Griesenbeck J, et al Structure of the initiation-competent RNA polymerase I and its implication for transcription. Nat Commun. 2016 7 15;7:12126 10.1038/ncomms12126 27418187 PMC4947174

[58] Baron U, Gossen M, Bujard H. Tetracycline-controlled transcription in eukaryotes: novel transactivators with graded transactivation potential. Nucleic Acids Res. 1997 7 15;25(14):2723–9. 9207017 10.1093/nar/25.14.2723 PMC146828

[59] Oakes M, Nogi Y, Clark MW, Nomura M. Structural alterations of the nucleolus in mutants of Saccharomyces cerevisiae defective in RNA polymerase I. Mol Cell Biol. 1993 4;13(4):2441–55. 8455621 10.1128/mcb.13.4.2441-2455.1993 PMC359565

[60] Trumtel S, Léger-Silvestre I, Gleizes PE, Teulières F, Gas N. Assembly and functional organization of the nucleolus: ultrastructural analysis of Saccharomyces cerevisiae mutants. Mol Biol Cell. 2000 6;11(6):2175–89. 10.1091/mbc.11.6.2175 10848637 PMC14911

[61] Merl J, Jakob S, Ridinger K, Hierlmeier T, Deutzmann R, Milkereit P, et al Analysis of ribosome biogenesis factor-modules in yeast cells depleted from pre-ribosomes. Nucleic Acids Res. 2010 5;38(9):3068–80. 10.1093/nar/gkp1244 20100801 PMC2875017

[62] Zaman S, Choudhury M, Jiang JC, Srivastava P, Mohanty BK, Danielson C, et al Mechanism of Regulation of Intrachromatid Recombination and Long-Range Chromosome Interactions in Saccharomyces cerevisiae. Mol Cell Biol. 2016 15;36(10):1451–63. 10.1128/MCB.01100-15 26951198 PMC4859694

[63] Breitkreutz A, Choi H, Sharom JR, Boucher L, Neduva V, Larsen B, et al A global protein kinase and phosphatase interaction network in yeast. Science. 2010 5 21;328(5981):1043–6. 10.1126/science.1176495 20489023 PMC3983991

[64] Krogan NJ, Cagney G, Yu H, Zhong G, Guo X, Ignatchenko A, et al Global landscape of protein complexes in the yeast Saccharomyces cerevisiae. Nature. 2006 3 30;440(7084):637–43. 10.1038/nature04670 16554755

[65] Beckouet F, Labarre-Mariotte S, Albert B, Imazawa Y, Werner M, Gadal O, et al Two RNA polymerase I subunits control the binding and release of Rrn3 during transcription. Mol Cell Biol. 2008 3;28(5):1596–605. 10.1128/MCB.01464-07 18086878 PMC2258765

[66] Zimmermann L, Stephens A, Nam S-Z, Rau D, Kübler J, Lozajic M, et al A Completely Reimplemented MPI Bioinformatics Toolkit with a New HHpred Server at its Core. J Mol Biol. 2018 7 20;430(15):2237–43. 10.1016/j.jmb.2017.12.007 29258817

[67] Söding J, Biegert A, Lupas AN. The HHpred interactive server for protein homology detection and structure prediction. Nucleic Acids Res. 2005 7 1;33(Web Server issue):W244–248. 10.1093/nar/gki408 15980461 PMC1160169

[68] Jantzen HM, Chow AM, King DS, Tjian R. Multiple domains of the RNA polymerase I activator hUBF interact with the TATA-binding protein complex hSL1 to mediate transcription. Genes Dev. 1992 10;6(10):1950–63. 1398072 10.1101/gad.6.10.1950

[69] McStay B, Frazier MW, Reeder RH. xUBF contains a novel dimerization domain essential for RNA polymerase I transcription. Genes Dev. 1991 11;5(11):1957–68. 1936987 10.1101/gad.5.11.1957

[70] Voit R, Schnapp A, Kuhn A, Rosenbauer H, Hirschmann P, Stunnenberg HG, et al The nucleolar transcription factor mUBF is phosphorylated by casein kinase II in the C-terminal hyperacidic tail which is essential for transactivation. EMBO J. 1992 6;11(6):2211–8. 1600946 10.1002/j.1460-2075.1992.tb05280.x PMC556688

[71] Maeda Y, Hisatake K, Kondo T, Hanada K, Song CZ, Nishimura T, et al Mouse rRNA gene transcription factor mUBF requires both HMG-box1 and an acidic tail for nucleolar accumulation: molecular analysis of the nucleolar targeting mechanism. EMBO J. 1992 10;11(10):3695–704. 1396565 10.1002/j.1460-2075.1992.tb05454.x PMC556829

[72] Ueshima S, Nagata K, Okuwaki M. Internal Associations of the Acidic Region of Upstream Binding Factor Control Its Nucleolar Localization. Mol Cell Biol. 2017 11 15;37(22). 10.1128/MCB.00218-17 PMC5660467 28874518

[73] Kihm AJ, Hershey JC, Haystead TA, Madsen CS, Owens GK. Phosphorylation of the rRNA transcription factor upstream binding factor promotes its association with TATA binding protein. Proc Natl Acad Sci U S A. 1998 12 8;95(25):14816–20. 9843972 10.1073/pnas.95.25.14816 PMC24532

[74] Lin C-Y, Tuan J, Scalia P, Bui T, Comai L. The cell cycle regulatory factor TAF1 stimulates ribosomal DNA transcription by binding to the activator UBF. Curr Biol CB. 2002 12 23;12(24):2142–6. 12498690 10.1016/s0960-9822(02)01389-1

[75] Zhai W, Comai L. A kinase activity associated with simian virus 40 large T antigen phosphorylates upstream binding factor (UBF) and promotes formation of a stable initiation complex between UBF and SL1. Mol Cell Biol. 1999 4;19(4):2791–802. 10082545 10.1128/mcb.19.4.2791 PMC84072

[76] Hannan KM, Brandenburger Y, Jenkins A, Sharkey K, Cavanaugh A, Rothblum L, et al mTOR-dependent regulation of ribosomal gene transcription requires S6K1 and is mediated by phosphorylation of the carboxy-terminal activation domain of the nucleolar transcription factor UBF. Mol Cell Biol. 2003 12;23(23):8862–77. 10.1128/MCB.23.23.8862-8877.2003 14612424 PMC262650

[77] Lin CH, Platt MD, Ficarro SB, Hoofnagle MH, Shabanowitz J, Comai L, et al Mass spectrometric identification of phosphorylation sites of rRNA transcription factor upstream binding factor. Am J Physiol Cell Physiol. 2007 5;292(5):C1617–1624. 10.1152/ajpcell.00176.2006 17182730

[78] O’Mahony DJ, Xie WQ, Smith SD, Singer HA, Rothblum LI. Differential phosphorylation and localization of the transcription factor UBF in vivo in response to serum deprivation. In vitro dephosphorylation of UBF reduces its transactivation properties. J Biol Chem. 1992 1 5;267(1):35–8. 1730600

[79] Stefanovsky VY, Pelletier G, Hannan R, Gagnon-Kugler T, Rothblum LI, Moss T. An immediate response of ribosomal transcription to growth factor stimulation in mammals is mediated by ERK phosphorylation of UBF. Mol Cell. 2001 11;8(5):1063–73. 11741541 10.1016/s1097-2765(01)00384-7

[80] Voit R, Kuhn A, Sander EE, Grummt I. Activation of mammalian ribosomal gene transcription requires phosphorylation of the nucleolar transcription factor UBF. Nucleic Acids Res. 1995 7 25;23(14):2593–9. 7651819 10.1093/nar/23.14.2593 PMC307079

[81] Voit R, Hoffmann M, Grummt I. Phosphorylation by G1-specific cdk-cyclin complexes activates the nucleolar transcription factor UBF. EMBO J. 1999 4 1;18(7):1891–9. 10.1093/emboj/18.7.1891 10202152 PMC1171274

[82] Blom N, Gammeltoft S, Brunak S. Sequence and structure-based prediction of eukaryotic protein phosphorylation sites. J Mol Biol. 1999 12 17;294(5):1351–62. 10.1006/jmbi.1999.3310 10600390

[83] Blom N, Sicheritz-Pontén T, Gupta R, Gammeltoft S, Brunak S. Prediction of post-translational glycosylation and phosphorylation of proteins from the amino acid sequence. Proteomics. 2004 6;4(6):1633–49. 10.1002/pmic.200300771 15174133

[84] Bierhoff H, Dundr M, Michels AA, Grummt I. Phosphorylation by casein kinase 2 facilitates rRNA gene transcription by promoting dissociation of TIF-IA from elongating RNA polymerase I. Mol Cell Biol. 2008 8;28(16):4988–98. 10.1128/MCB.00492-08 18559419 PMC2519707

[85] Panova TB, Panov KI, Russell J, Zomerdijk JCBM. Casein kinase 2 associates with initiation-competent RNA polymerase I and has multiple roles in ribosomal DNA transcription. Mol Cell Biol. 2006 8;26(16):5957–68. 10.1128/MCB.00673-06 16880508 PMC1592790

[86] Engel C, Neyer S, Cramer P. Distinct Mechanisms of Transcription Initiation by RNA Polymerases I and II. Annu Rev Biophys. 2018 5 20;47:425–46. 10.1146/annurev-biophys-070317-033058 29792819

[87] Ausubel FM. Current Protocols in Molecular Biology [Internet]. John Wiley & Sons; 1994. (Current Protocols in Molecular Biology). Available from: https://books.google.de/books?id=20EbAQAAMAAJ

[88] Green MR, Sambrook J. Molecular Cloning: A Laboratory Manual (Fourth Edition): Three-volume set. 4th edition Cold Spring Harbor, N.Y: Cold Spring Harbor Laboratory Press; 2012. 2028 p.

[89] Longtine MS, McKenzie A, Demarini DJ, Shah NG, Wach A, Brachat A, et al Additional modules for versatile and economical PCR-based gene deletion and modification in Saccharomyces cerevisiae. Yeast Chichester Engl. 1998 7;14(10):953–61. 10.1002/(SICI)1097-0061(199807)14:10<953::AID-YEA293>3.0.CO;2-U 9717241

[90] Laemmli UK. Cleavage of structural proteins during the assembly of the head of bacteriophage T4. Nature. 1970 8 15;227(5259):680–5. 5432063 10.1038/227680a0

[91] Towbin H, Staehelin T, Gordon J. Electrophoretic transfer of proteins from polyacrylamide gels to nitrocellulose sheets: procedure and some applications. Proc Natl Acad Sci U S A. 1979 9;76(9):4350–4. 388439 10.1073/pnas.76.9.4350 PMC411572

[92] Allefs JJ, Salentijn EM, Krens FA, Rouwendal GJ. Optimization of non-radioactive Southern blot hybridization: single copy detection and reuse of blots. Nucleic Acids Res. 1990 5 25;18(10):3099–100. 2349131 10.1093/nar/18.10.3099 PMC330882

[93] Knop M, Siegers K, Pereira G, Zachariae W, Winsor B, Nasmyth K, et al Epitope tagging of yeast genes using a PCR-based strategy: more tags and improved practical routines. Yeast Chichester Engl. 1999 7;15(10B):963–72. 10.1002/(SICI)1097-0061(199907)15:10B<963::AID-YEA399>3.0.CO;2-W 10407276

[94] Berger I, Fitzgerald DJ, Richmond TJ. Baculovirus expression system for heterologous multiprotein complexes. Nat Biotechnol. 2004 12;22(12):1583–7. 10.1038/nbt1036 15568020

[95] Fitzgerald DJ, Berger P, Schaffitzel C, Yamada K, Richmond TJ, Berger I. Protein complex expression by using multigene baculoviral vectors. Nat Methods. 2006 12;3(12):1021–32. 10.1038/nmeth983 17117155

[96] Hierlmeier T, Merl J, Sauert M, Perez-Fernandez J, Schultz P, Bruckmann A, et al Rrp5p, Noc1p and Noc2p form a protein module which is part of early large ribosomal subunit precursors in S. cerevisiae. Nucleic Acids Res. 2013 1;41(2):1191–210. 10.1093/nar/gks1056 23209026 PMC3553968

[97] Hamperl S, Brown CR, Perez-Fernandez J, Huber K, Wittner M, Babl V, et al Purification of specific chromatin domains from single-copy gene loci in Saccharomyces cerevisiae. Methods Mol Biol Clifton NJ. 2014;1094:329–41. 10.1007/978-1-62703-706-8_26 24163000

