# Supplementary figures and images for: The C-terminal region of Net1 is an activator of RNA polymerase I transcription with conserved features from yeast to human
Source: PLoS Genet. 2019 Feb 25;15(2):e1008006. doi: 10.1371/journal.pgen.1008006 (PMC6415870; doi:10.1371/journal.pgen.1008006)

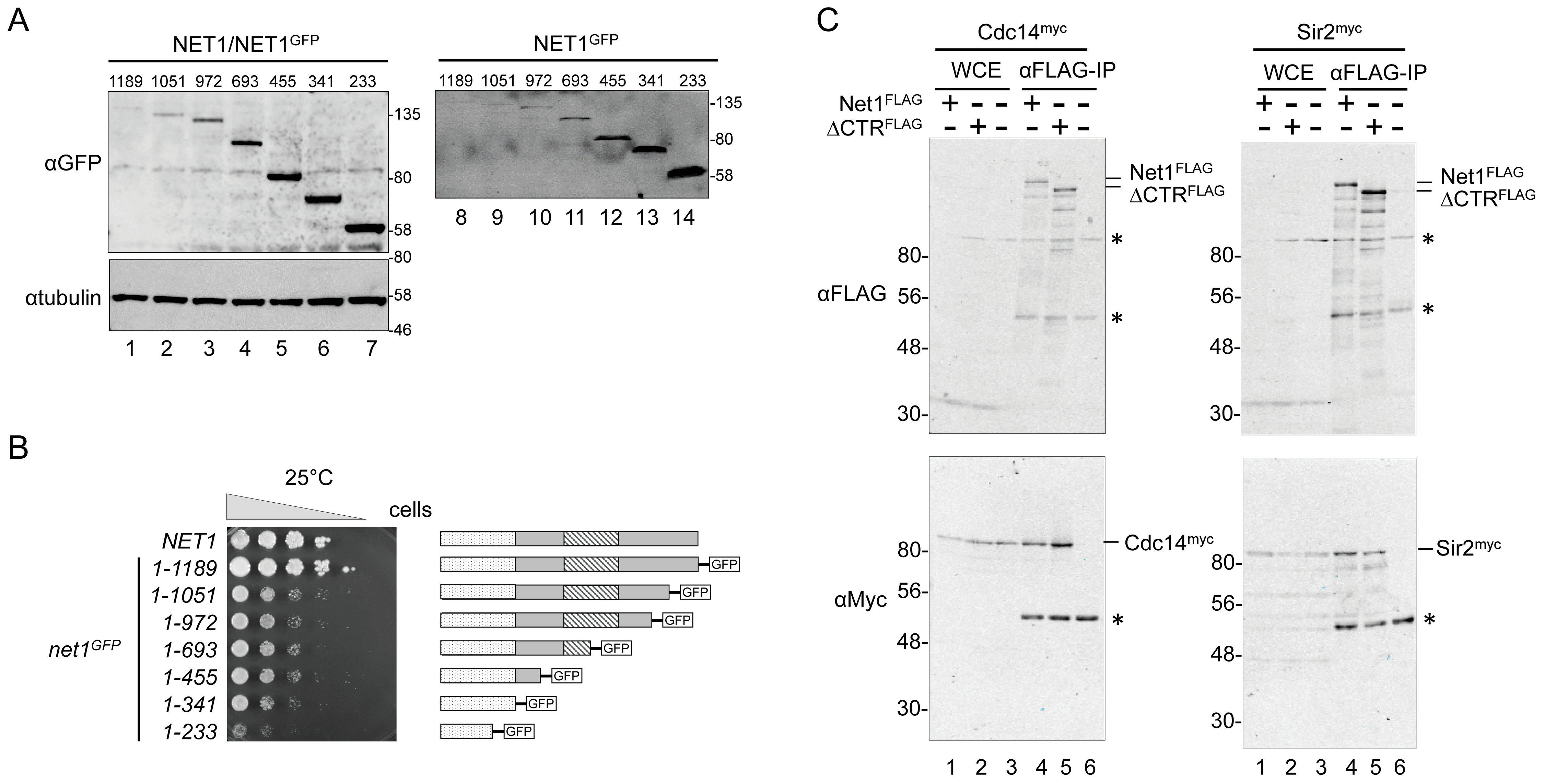

Supplement: S1 Fig — A) WCEs of diploid yeast strains (W13533, W13534, W13535, W13536, W13537, W13538, W13539, W11979) (lanes 1–7), or the respective haploid progenies (lanes 8–14), expressing the indicated GFP fusion proteins, were prepared and subjected to western blot analysis with anti-GFP antibody (αGFP, upper panel) and anti-tubulin antibody (αtubulin, lower panel) as described in the legend to Fig 2C. B) Diploid yeast strains (W13533, W13534, W13535, W13536, W13537, W13538, W13539, W11979) were sporulated yielding haploid progenies carrying alleles for expression of the indicated GFP-fusion proteins. Cartoons of the Net1-GFP proteins are depicted on the right according to Fig 1B. Serial dilutions of cell suspensions of the haploid strains and a control strain K699 carrying a NET1 wild-type allele were spotted on XYD plates and incubated at 25°C for 2d before a photograph was taken. C) Pictures of the full-size membranes shown in Fig 2C (see legend to this figure for more information). An asterisk on the right marks bands which are the result of cross-hybridization of antibodies with cellular proteins in WCEs and chains of antibody used for the IPs. (TIF) [file pgen.1008006.s001.tif]

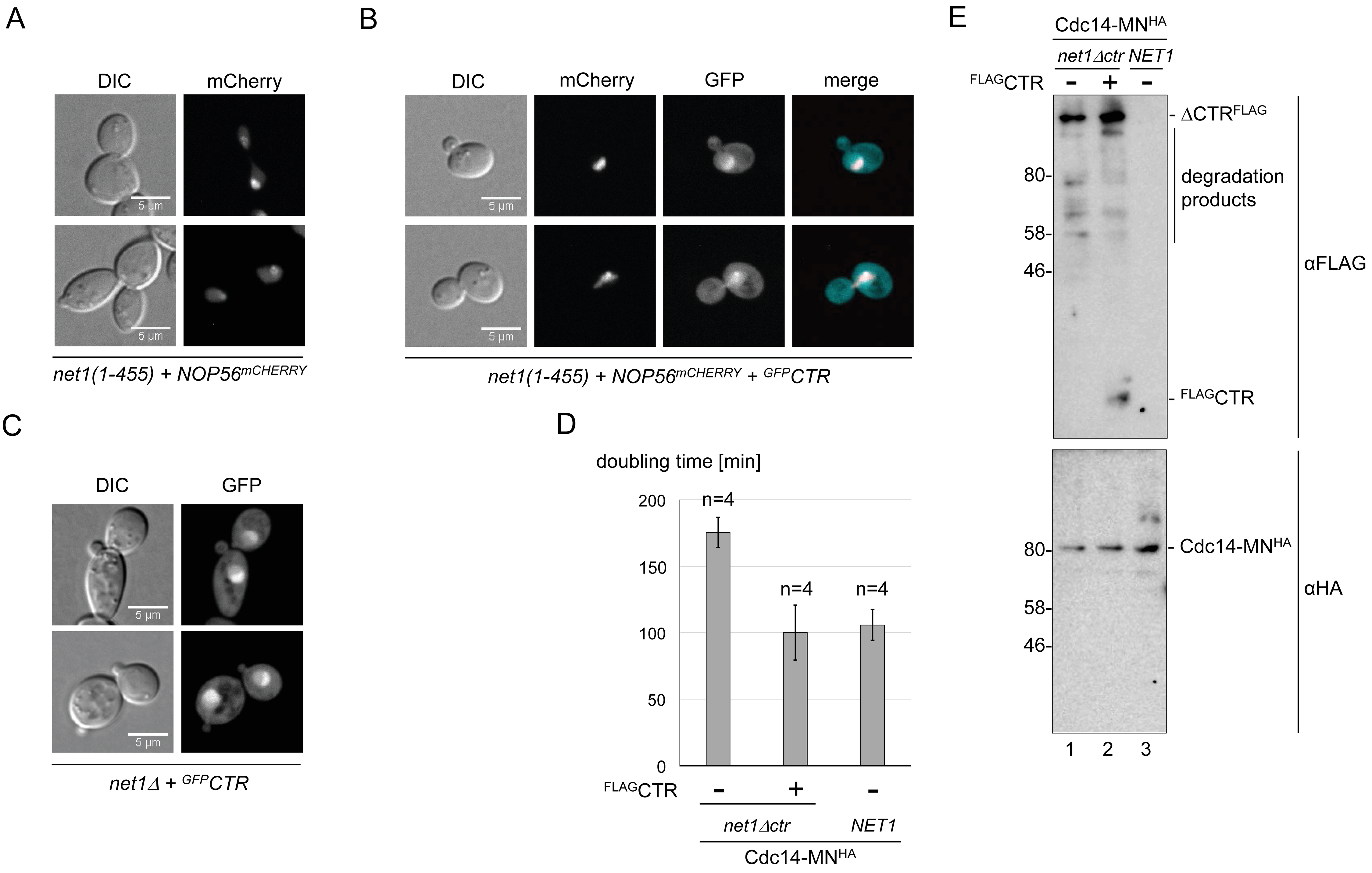

Supplement: S2 Fig — A-C) CTR expression in trans re-establishes wild-type cell morphology and Nop56 nucleolar localization in net1(1–455) strains and has a preferential nucleolar localization in net1Δ strains. A,B) The diploid yeast strain W15406 was sporulated, yielding haploid progenies carrying a net1(1–455) allele, and expressing Nop56mCherry in the absence (A) and presence of GFPCTR (B). Haploid strains were subjected to live cell fluorescence microscopy as described in the Legend to Fig 2D. C) The diploid yeast strain W12509 was sporulated, yielding haploid progenies carrying a net1Δ allele, and expressing GFPCTR. The haploid strain was subjected to live cell fluorescence microscopy as described in the Legend to Fig 2D. D,E) FLAGCTR expression suppresses the growth defect of net1Δctr strains and does not alter expression levels of Net1ΔCTRFLAG Haploid yeast strains (y3058, y3068, y3250), carrying a NET1 or a net1Δctr allele, and expressing Cdc14-MNHA in the absence or presence of a chromosomally integrated expression cassette for FLAGCTR were subjected to growth and western blot analyses. D) Growth analyses in liquid culture were performed as described in the legend to Fig 2B. E) WCEs were prepared and subjected to western blot analysis as described in the legend to Fig 2C, using anti-FLAG antibody (αFLAG, top panel), or anti-HA antibody (αHA, bottom panel). The positions of tagged proteins on the membrane, as well as of degradation products of Net1ΔCTRFLAG are indicated on the right. (TIF) [file pgen.1008006.s002.tif]

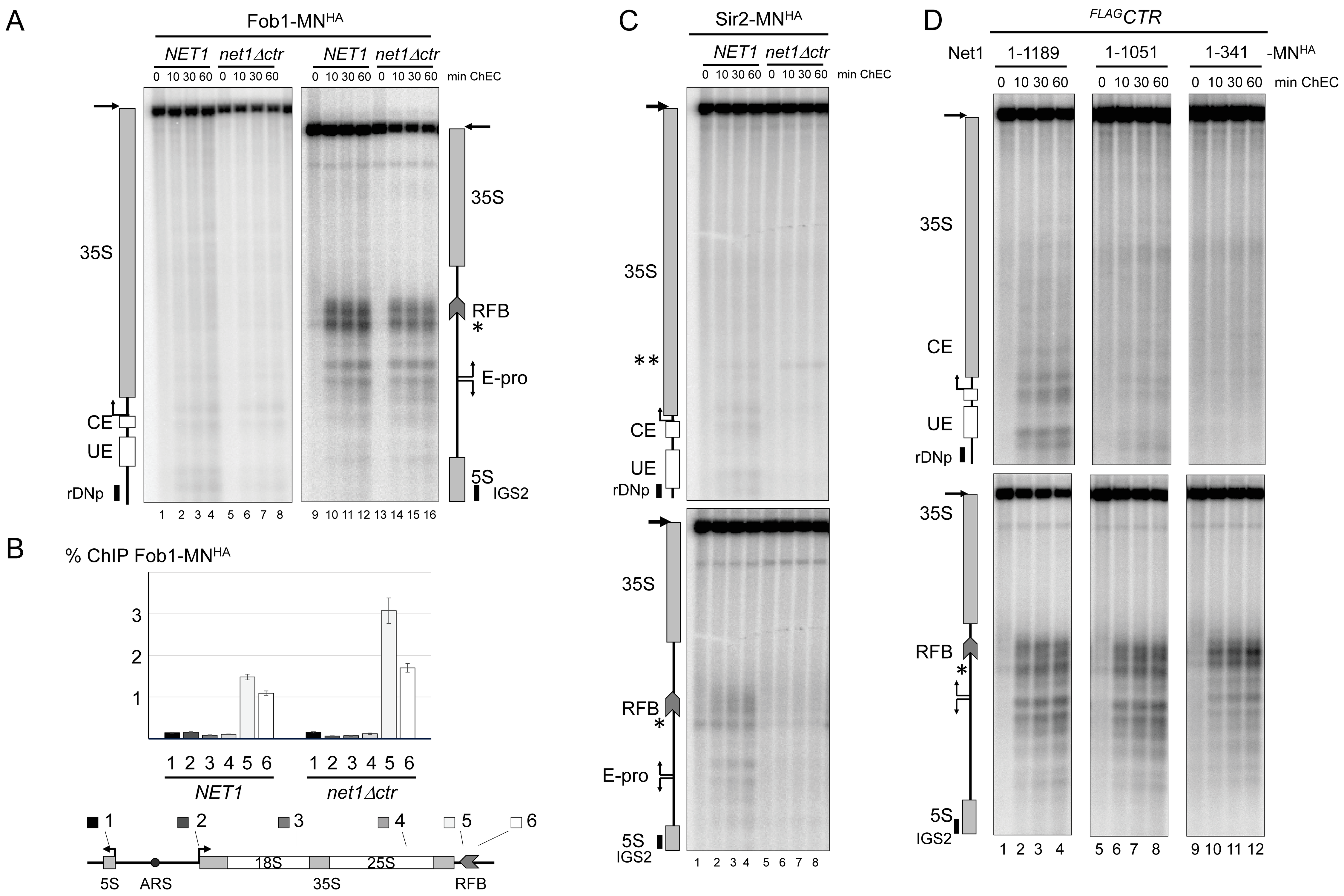

Supplement: S3 Fig — Haploid yeast strains were subjected to ChEC (A,C,D) and ChIP (B) analyses as described in the legend to Fig 4. A-C) Association of Fob1 and Sir2 with rDNA is impaired in net1Δctr strains A,B) ChEC and ChIP analyses with strains y952, and y3040, carrying a NET1 or a net1Δctr allele, and expressing Fob1-MNHA. C) ChEC analyses with strains y1450, and y2966, carrying a NET1 or a net1Δctr allele, and expressing Sir2-MNHA. Two asterisks label the position of a fragment which was dependent upon the addition of calcium to the crude nuclei. This fragment was unrelated to Sir2-MNHA since it was also observed in strains not expressing any MN fusion protein (not shown). D) C-terminal truncation of Net1 abolishes association with the 35S rDNA promoter and cannot be restored upon expression of FLAGCTR ChEC analyses with yeast strains (y3157; y3164; y3145), expressing Net1-MNHA, Net1ΔCTR-MNHA, or Net1(1–341)-MNHA from the endogenous NET1 locus, and FLAGCTR from a chromosomally integrated cassette. (TIF) [file pgen.1008006.s003.tif]

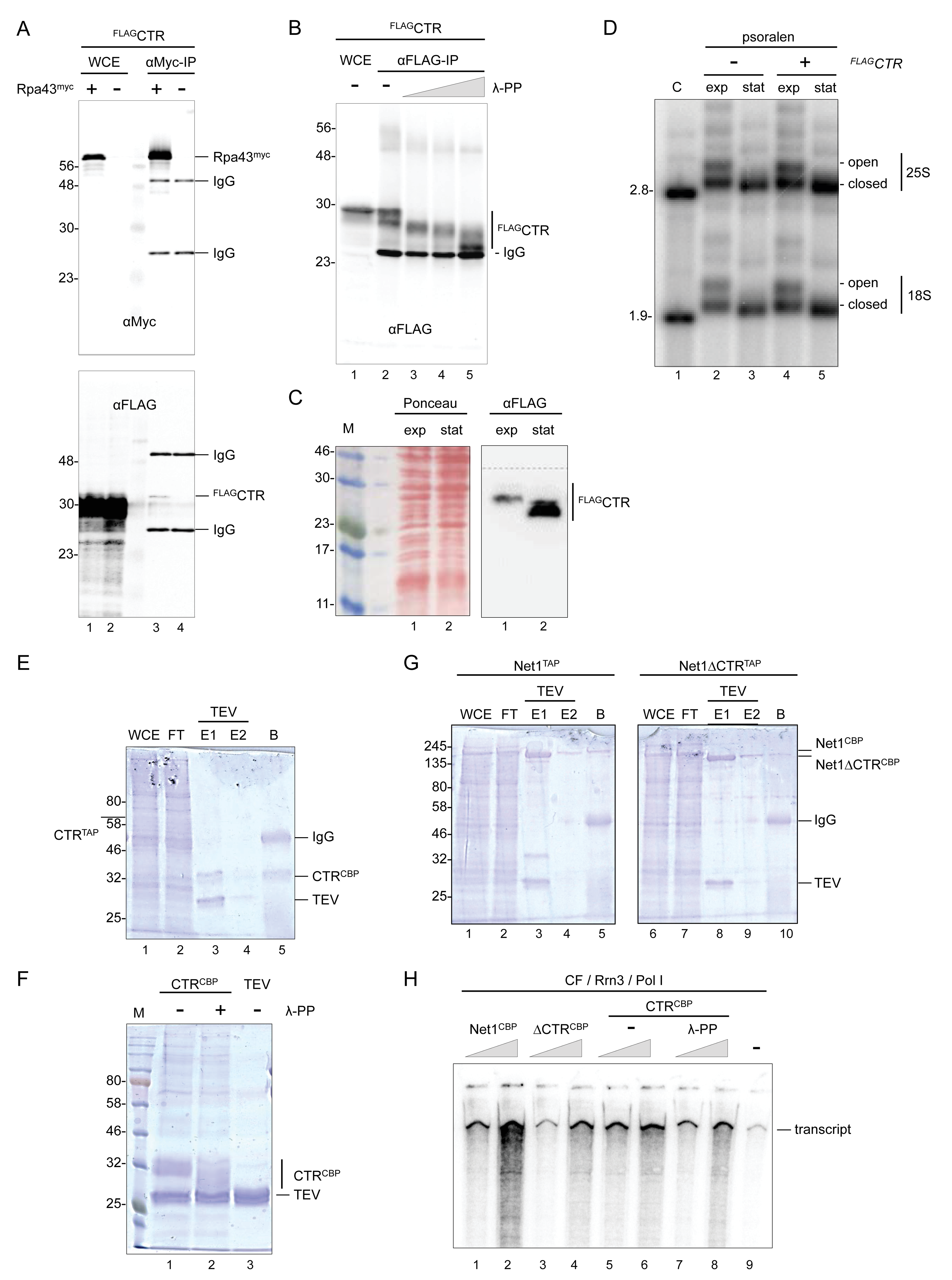

Supplement: S4 Fig — A-C) The CTR is differentially phosphorylated in exponentially growing and stationary yeast cells in vivo A-C) Fluorographs and Ponceau staining of the full-size membranes shown in Fig 6A–6C (see legend to these figures for more information). D) Haploid yeast strain y3739 expressing FLAGCTR was cultured in YPD. Different samples were withdrawn at exponential (exp) and stationary phase (stat), and either used for protein extraction (Fig 6C; S4C Fig) or treated with formaldehyde for psoralen crosslinking analysis. Crude nuclear extracts were prepared from formaldehyde treated cells, which were subjected to the psoralen crosslinking procedure. DNA was isolated, digested with EcoRI, separated by native agarose gel and subjected to Southern blot analyses with probe “3.5kb rDNA”. Positions of rDNA fragments derived from Pol I transcribed “open” 35S rRNA genes and nucleosomal “closed” 35S rRNA genes are depicted on the right. An autoradiography of the Southern blot membrane is shown. Lanes 1 and 2 show the analysis of a control experiment with strain y3725, which did not express FLAGCTR. Lane C included genomic DNA digested with EcoRI, isolated from a strain which was not subjected to psoralen crosslinking but mock treated in parallel with the other strains. The sizes of two DNA fragments in kb spanning either parts of the 18S or the 25S rDNA transcribed by Pol I and visualized by probe “3.5kb rDNA” are depicted on the left. E-H) Recombinant Net1, Net1DCTR and CTR purified from insect cells stimulate promoter-dependent Pol I transcription in vitro, and CTR-mediated stimulation of in vitro transcription is reduced upon λ-protein phosphatase treatment E,G) Purification of recombinant Net1TAP variants from bacmid infected Sf9 cells was performed as described in the legend to Fig 6D. Proteins contained in samples of the whole-cell extract (WCE, 0.06% of total), the flow through (FT, 0.06%), elution 1 and 2 after cleavage with TEV protease (E1, E2, 20%), and beads after elution [file pgen.1008006.s004.tif]

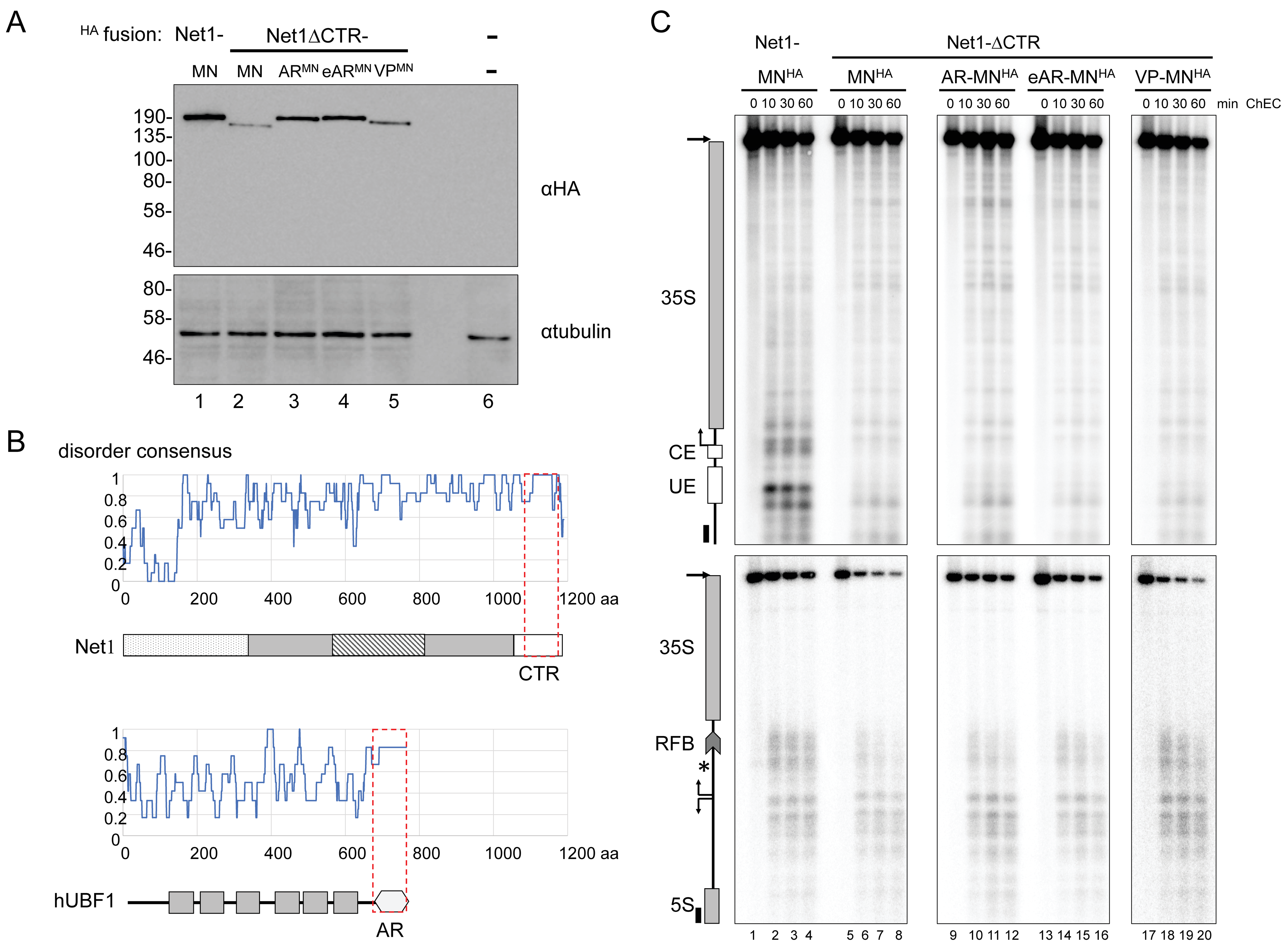

Supplement: S5 Fig — A) Chemiluminescence on the full-size membranes shown in Fig 7C. B) Secondary structure prediction by the Disorder Prediction Meta-Server (http://www-nmr.cabm.rutgers.edu/bioinformatics/disorder/) for yeast Net1 (amino acids (aa) 1–1189; GI: 1023942937) and hUBF1 (aa 1–764; GI: 7657671). The disorder consensus model was created using 6 public structure prediction tools. The Disorder consensus (1 = disordered, 0 = structured) is plotted against the amino acid sequence of full-length yeast Net1 (top), and full length hUBF1. Scaled cartoons of Net1 and hUBF1 are depicted below the respective graphs with symbols described in the legends to Fig 1B and Fig 7A, respectively. A red dotted rectangle frames the regions shown in the pairwise sequence alignment in Fig 7A. C) Haploid yeast strains (y4066; y4068; y4070; y4072; y4074) expressing the indicated MNHA fusion proteins were subjected to ChEC analyses as described in Fig 4. (TIF) [file pgen.1008006.s005.tif]
